# The Polymorphic Pseudokinase ROP5 Controls Virulence in *Toxoplasma gondii* by Regulating the Active Kinase ROP18

**DOI:** 10.1371/journal.ppat.1002992

**Published:** 2012-11-08

**Authors:** Michael S. Behnke, Sarah J. Fentress, Mona Mashayekhi, Lucy X. Li, Gregory A. Taylor, L. David Sibley

**Affiliations:** 1 Department of Molecular Microbiology, Washington University School of Medicine, St. Louis, Missouri, United States of America; 2 Department of Pathology and Immunology, Washington University School of Medicine, St. Louis, Missouri, United States of America; 3 Departments of Medicine, Molecular Genetics and Microbiology, and Immunology, Division of Geriatrics, and Center for the Study of Aging and Human Development, Duke University Medical Center, Durham, North Carolina, United States of America; 4 Geriatric Research, Education, and Clinical Center, VA Medical Center, Durham, North Carolina, United States of America; Cornell University, United States of America

## Abstract

Secretory polymorphic serine/threonine kinases control pathogenesis of *Toxoplasma gondii* in the mouse. Genetic studies show that the pseudokinase ROP5 is essential for acute virulence, but do not reveal its mechanism of action. Here we demonstrate that ROP5 controls virulence by blocking IFN-γ mediated clearance in activated macrophages. ROP5 was required for the catalytic activity of the active S/T kinase ROP18, which phosphorylates host immunity related GTPases (IRGs) and protects the parasite from clearance. ROP5 directly regulated activity of ROP18 *in vitro*, and both proteins were necessary to avoid IRG recruitment and clearance in macrophages. Clearance of both the Δ*rop5* and Δ*rop18* mutants was reversed in macrophages lacking Irgm3, which is required for IRG function, and the virulence defect was fully restored in Irgm3^−/−^ mice. Our findings establish that the pseudokinase ROP5 controls the activity of ROP18, thereby blocking IRG mediated clearance in macrophages. Additionally, ROP5 has other functions that are also Irgm3 and IFN-γ dependent, indicting it plays a general role in governing virulence factors that block immunity.

## Author Summary

The ability of microorganisms to cause disease in their hosts is often mediated by proteins that are secreted by the pathogen into the host cell as a means of disarming host signaling. Previous studies with the protozoan parasite *Toxoplasma gondii* have revealed that secretion of parasite protein kinases into the host cell mediates virulence in mouse, a natural host for transmission. Curiously, some of these virulence factors are active protein kinases, while other related pseudokinases lack enzymatic activity; hence, it was unclear how they functioned in promoting virulence. In the present work we demonstrate that ROP5, an inactive member of this protein kinase family, regulates the active protein kinase ROP18, which normally prevents clearance of the parasite in interferon-activated macrophages. Allosteric regulation of enzymes is a common theme in biology, but this is the first example of such a mechanism regulating a pathogen virulence factor. The potential advantage of such a layered process is that it might allow greater temporal or spatial control and perhaps protect the parasite from disabling strategies by the host.

## Introduction

*Toxoplasma gondii* is an obligate intracellular parasite that infects a wide range of vertebrate animal hosts and causes zoonotic infection in humans, leading to potentially severe congenital infections and risk of reactivation in immunocompromised patients [1]. In North America and Europe, *T. gondii* exists as four distinct clonal lineages that show marked virulence differences in laboratory mice, which serve as a model for infection [2], [3]. Forward genetic analyses have been used to map the genes responsible for virulence in laboratory mice [4], [5]. Remarkably, this complex trait is largely mediated by a few members of a large family of polymorphic serine threonine (S/T) protein kinases secreted from rhoptries (ROP) into the host cell during invasion [6], [7]. The ROP kinase family consists of ∼20 active members, as well as a similar number of putative pseudokinases that are predicted to lack kinase activity [8]. The structures of several ROP pseudokinases reveal they contain a typical kinase fold and yet they are structurally and phylogenetically diverse [9], [10].

Most strains of *T. gondii* survive within naïve macrophages; however, when previously activated by exposure to IFN-γ macrophages acquire the ability to kill or inhibit parasites [11]. During primary infection, inflammatory monocytes are recruited to the site of infection where they are critical for control of intracellular *T. gondii* [12], [13]. Macrophages control *T. gondii* through induction of iNOS, which leads to stasis [14], reactive oxygen intermediates, which leads to killing of opsonized parasites [15], and upregulation of immunity related GTPases (IRGs), which destroy intracellular parasites [16], [17]. Recruitment of IRG effectors to the parasite containing vacuole results in destruction of the parasite residing within it [18], [19]. Compared to most mammals, the IRG gene family is highly amplified in rodents [20], where it plays a major role in natural resistance to *T. gondii* [21], [22].

Not all strains of *T. gondii* are susceptible to clearance in IFN-γ-activated macrophages: highly mouse virulent type I parasites resist IRG recruitment and consequently avoid clearance, while intermediate virulent type II and avirulent type III parasites are unable to block IRG recruitment and are destroyed [14], [23]. Recent studies have revealed the mechanism for this escape: the S/T kinase ROP18 phosphorylates a number of IRGs on key threonine residues in switch region I of the GTPase domain, thereby preventing assembly on the vacuole and blocking clearance in activated macrophages [24], [25]. The IRG system is also dependent on the autophagy protein Atg5, although the molecular basis for this requirement remains unclear [23], [26].

Genetic mapping studies have also implicated the pseudokinase ROP5 in acute virulence [27], [28], a result that is unlikely to be due to kinase activity as ROP5 lacks a key catalytic residue and binds ATP in an unconventional manner [29]. Comparison of several pair-wise genetic crosses suggest that ROP5 interacts with ROP18 [27]; however, the molecular basis for the dramatic effects of ROP5 on virulence remains uncertain. Pseudokinases in other systems have recently been shown to perform regulatory roles [30], raising a similar possibility for ROP5. Herein, we explore the mechanism of action for ROP5 and demonstrate that it controls ROP18 activity, while also serving a separate and essential role in acute virulence.

## Results

### ROP5 deficient parasites show limited expansion *in vivo*

**Figure 1:**
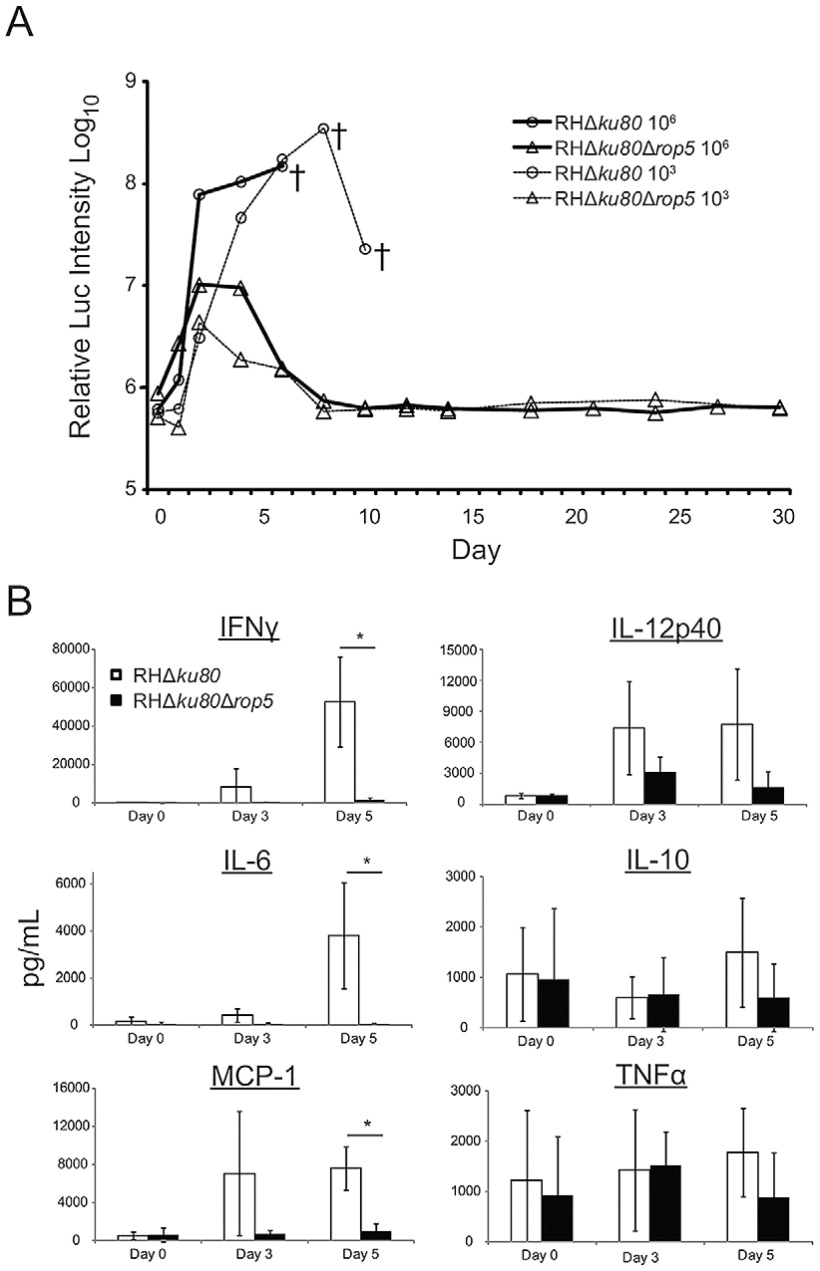
ROP5 deficient parasites are controlled in wild type mice and trigger a cytokine response proportional to the parasite load. (A) *In vivo* parasite growth was monitored using luciferase expressing wild type (RHΔ*ku80*) or ROP5 deficient (RHΔ*ku80*Δ*rop5*) parasites. CD-1 mice were i.p. injected with either 10^6^ or 10^3^ parasites and imaged on indicated days. † denotes one or more deaths. Mean values shown per group (n = 5). Representative of 2 experiments with similar outcomes. (B) Analysis of cytokines in serum of infected mice used in (A) (10^6^ inoculum) on days 0 (uninfected), 3, and 5. The levels of IFN-γ, IL-6, MCP-1, IL-10, and TNF-α were determined using a Cytometric Bead Array analyzed on a FACS Canto. IL-12p40 was measured by ELISA. Mean ± S.D., n = 3 animals per group. Student's *t* test * *P*<0.01. Representative of 2 experiments with similar outcomes.

Previous studies have shown that ROP5 deficient (RHΔ*ku80*Δ*rop5*) parasites grow normally *in vitro*, but are highly attenuated in laboratory mice [27]; however, the molecular basis of this phenotype is not understood. To examine growth *in vivo*, CD-1 outbred mice were infected with either wild type (RHΔ*ku80)* or ROP5 deficient (RHΔ*ku80*Δ*rop5*) parasites expressing luciferase and growth was followed over time. Virulent RHΔ*ku80* parasites expanded rapidly, as detected by luciferase activity, until the mice succumbed to infection between days 6–8 (Figure 1A, Figure S1). In contrast, ROP5 deficient (RHΔ*ku80*Δ*rop5*) parasites expanded normally for the first few days and then dramatically decreased by the end of the first week, resulting in survival of the mice (Figure 1, Figure S1).

To examine innate immune responses, we measured inflammatory cytokines in serum during the first week post infection. The levels of IFN-γ, IL-6, and MCP-1 increased at day 3 and were all significantly higher at day 5 (*P*≤0.01) in mice infected with wild type (RHΔ*ku80*) parasites as compared to ROP5 deficient (RHΔ*ku80*Δ*rop5*) parasites (Figure 1A, B). Consistent with a rise in IFN-γ, we also observed an increase in IL-12p40 at day 3 and 5 in mice infected with wild type (RHΔ*ku80*) parasites. In contrast, IL-10 and TNFα were essentially unchanged. Overall, changes in cytokine levels appeared to track closely with parasite burden.

To test whether ROP5 modulates host cell transcription, we infected human foreskin fibroblasts (HFF) with either wild type (RHΔ*ku80*) or ROP5 deficient (RHΔ*ku80*Δ*rop5*) parasites and examined gene expression using the Affymetrix HG-U113A_2 Human Array. There were no significant differences in gene expression in HFF infected with wild type (RHΔ*ku80*) or ROP5 deficient (RHΔ*ku80*Δ*rop5*) parasites (NCBI GEO record GSE32104), indicating ROP5 does not directly modulate host gene expression, at least under the conditions tested *in vitro*.

### IFN-γ signaling is required for clearance of ROP5 deficient parasites

**Figure 2:**
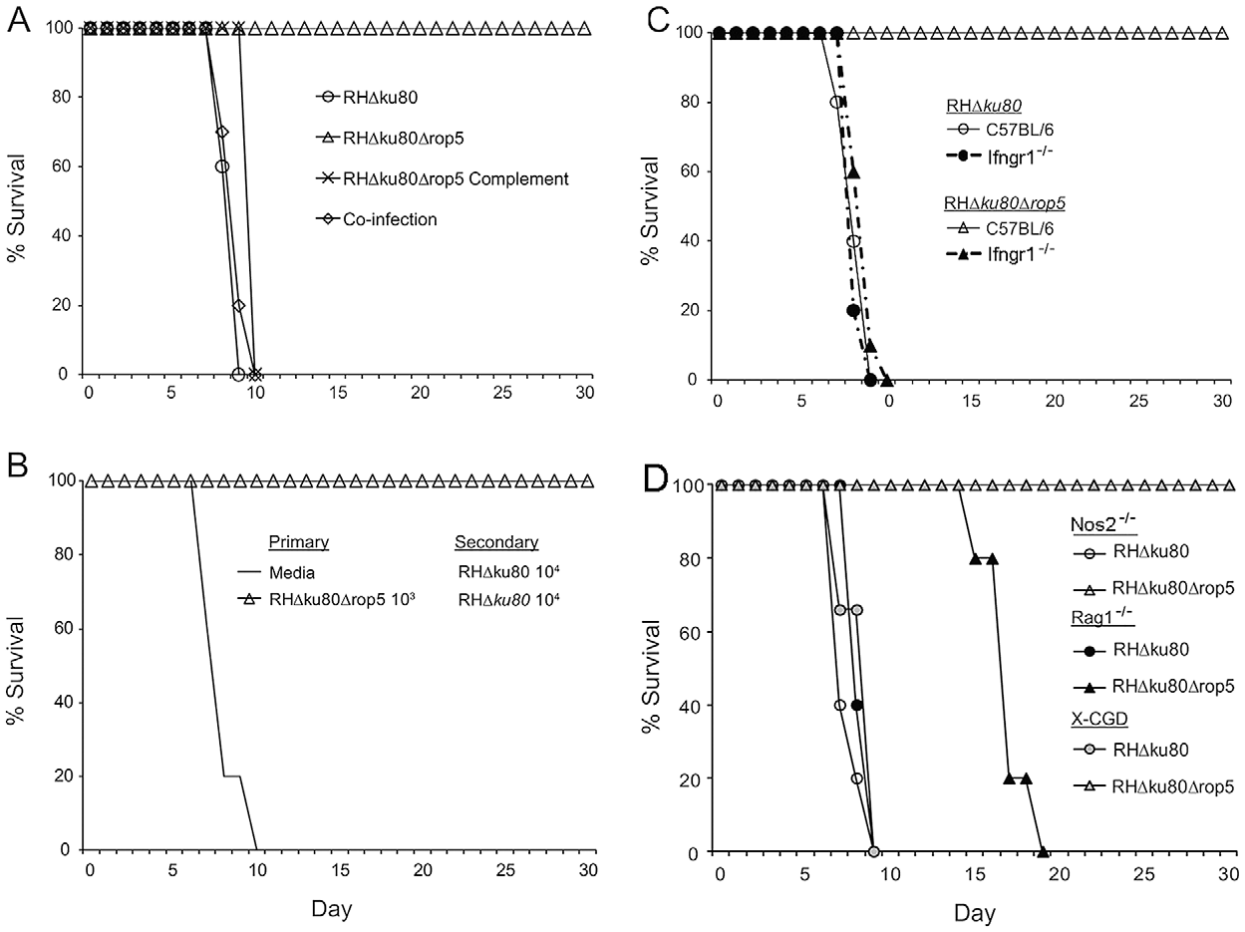
ROP5 deficient parasites vaccinate against lethal challenge and IFN-γ signaling is required for control of Δ*rop5* parasites. (A) CD-1 mice were separately injected i.p. with 10^3^ wild type (RHΔ*ku80*), ROP5 deficient (RHΔ*ku80*Δ*rop5*) or ROP5 complemented (RHΔ*ku80*Δ*rop5*Complement) parasites, or co-infected with 10^3^ each of wild type (RHΔ*ku80*) and ROP5 deficient (RHΔ*ku80*Δ*rop5*) parasites. Survival was followed for 30 days. Mean values shown per group (n = 5). Representative of 2 experiments with similar outcomes. (B) CD-1 mice were inoculated with either media alone or 10^3^ ROP5 deficient (RHΔ*ku80*Δ*rop5*) parasites. After 30 days, mice were challenged with 10^4^ wild type (RHΔ*ku80*) parasites and survival followed for 30 days. Mean values shown per group (n = 5). Representative of 2 experiments with similar outcomes. (C) C57BL/6 control or Ifngr1^−/−^ mice were i.p. injected with 10^3^ wild type (RHΔ*ku80*) or ROP5 deficient (RHΔ*ku80*Δ*rop5*) parasites and survival was followed for 30 days. Mean values shown per group (n = 5). Representative of 3 experiments with similar outcomes. (D) Nos2^−/−^, Rag1^−/−^, or X-CGD mice were injected i.p. with 10^3^ wild type (RHΔ*ku80*) or ROP5 deficient (RHΔ*ku80*Δ*rop5*) parasites and survival was followed for 30 days. Mean values shown per group (n = 5), except X-CGD mice (RHΔ*ku80* n = 3 and RHΔ*ku80*Δ*rop5* n = 4). Representative of 2 experiments with similar outcomes.

The failure of ROP5 deficient parasites to expand *in vivo* could emanate from some alteration in the immune response. For example, if ROP5 were normally immunosuppressive, in its absence, a stronger or more potent immune response might provide more effective control of infection. To test whether ROP5 deficient parasites lack a normally suppressive function, mice were infected separately, or coinfected with wild type (RHΔ*ku80*) and ROP5 deficient (RHΔ*ku80*Δ*rop5*) parasites, and followed for 30 days to assess survival. Consistent with previous studies, mice infected with wild type (RH*Δku80*) or ROP5 complemented (RH*Δku80Δrop5* Complement) parasites succumbed in 9–10 days (Figure 2A). Although ROP5 deficient parasites failed to cause lethal infection, coinfection with wild type parasites led to rapid death of all mice by day 10 (Figure 2A). Moreover, mice immunized with ROP5 deficient parasites were able to generate a normal adaptive immune response and survive a secondary challenge with a normally lethal dose of wild type parasites (Figure 2B). These findings indicate that it is unlikely that ROP5 is globally immunosuppressive.

Alternatively, it was possible that ROP5 deficient parasites were metabolically restricted *in vivo*, similar to the previously reported pyrimidine biosynthesis mutants (i.e. *cpsII* mutants), which are unable to propagate *in vivo* due limitations in uracil for salvage [31]. *cpsII* mutants fail to expand in both wild type and Ifng^−/−^ mice [31], consistent with their metabolic limitation. To determine if ROP5 deficient (RHΔ*ku80*Δ*rop5*) parasites were controlled by an IFN-γ-dependent mechanism, we tested the virulence of ROP5 deficient (RHΔ*ku80*Δ*rop5*) parasites in Ifngr1^−/−^ mice, which are unable to respond to IFN-γ and hence, are highly susceptible to toxoplasmosis [32]. Ifngr1^−/−^ mice were completely susceptible to infection with ROP5 deficient parasites, succumbing in the same time frame as wild type C57BL/6 or Ifngr1^−/−^ mice infected with virulent wild type parasites (Figure 2C). To further clarify the role of ROP5 in immune evasion we tested the virulence of ROP5 deficient (RHΔ*ku80*Δ*rop5*) parasites in mice lacking the inducible nitric oxide synthase (iNOS) (Nos2^−/−^mice), the superoxide-generating NADPH-oxidase gp91^phox^ (X-CGD mice), or the recombination activating gene 1 (Rag1^−/−^ mice), important for B and T cell function. iNOS^−/−^ and X-CGD mice survived infection with ROP5 deficient (RHΔ*ku80*Δ*rop5*) parasites (Figure 2D) and did not present symptoms of illness or weight loss (data not shown), similar to C57BL/6 control mice (Figure 2C). Although Rag1^−/−^ mice succumbed to ROP5 deficient (RHΔ*ku80*Δ*rop5*) infection, death was delayed compared to wild type (RHΔ*ku80*) infection (Figure 2D).

Collectively these findings indicate that IFN-γ signaling is necessary for controlling ROP5 deficient parasites and suggest that ROP5 is critical to the parasites' ability to resist innate immune effectors generated by the IFN-γ response.

### Inflammatory monocytes are recruited to the site of infection in mice infected with ROP5 deficient parasites

**Figure 3:**
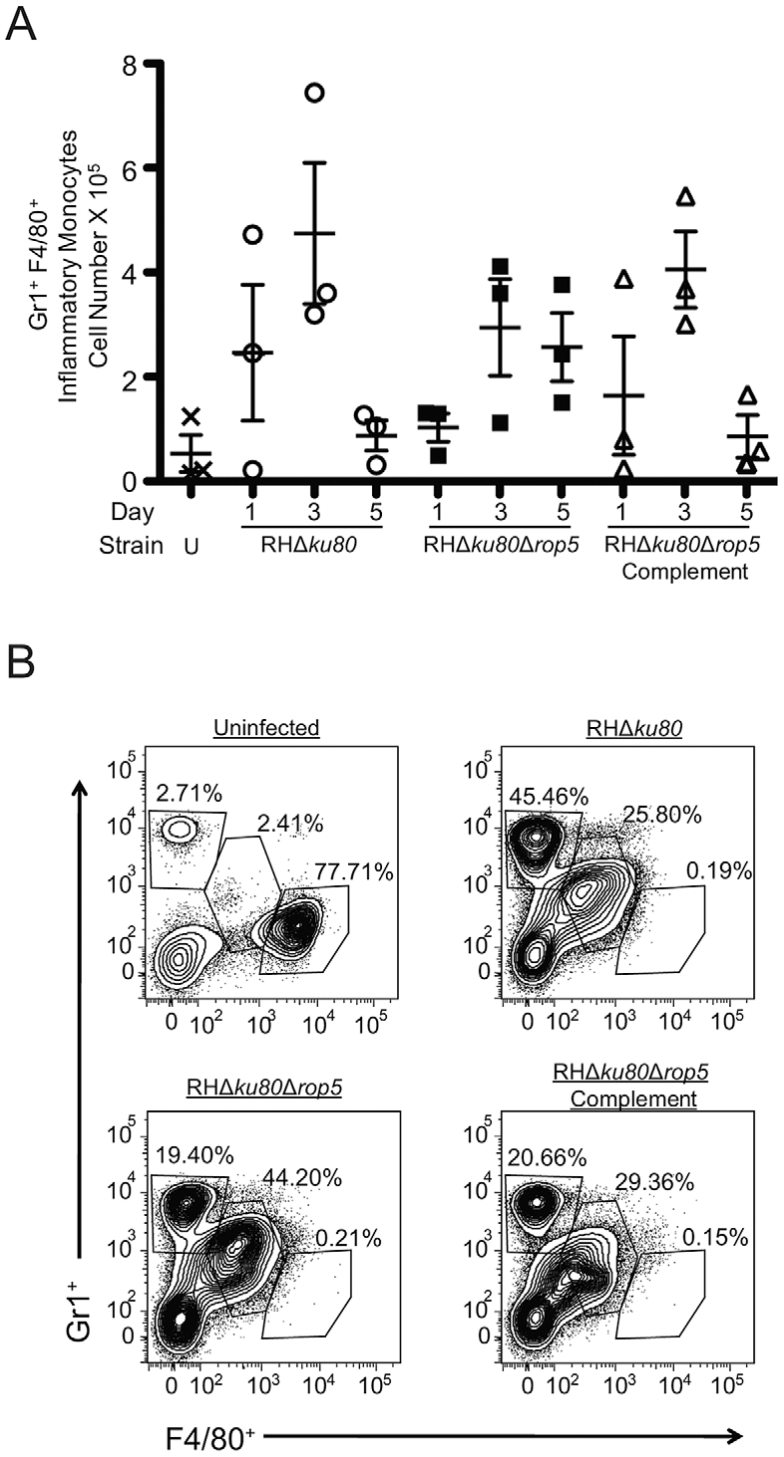
Gr1^+^ F4/80^+^ inflammatory monocytes are recruited to the peritoneum after infection with wild type or ROP5 deficient parasites. (A) CD-1 mice were infected with 10^3^ wild type (RHΔ*ku80*), ROP5 deficient (RHΔ*ku80*Δ*rop5*), or ROP5 complemented (RHΔ*ku80*Δ*rop5*Complement) parasites and the number of Gr1^+^ F4/80^+^ cells was determined by cell surface staining and FACS. Mean ± S.E.M., n = 3 animals per group. (B) FACS plots of Gr1 and F4/80 gated cells from representative animals at day 3 postinfection in (A). Top left gate represents neutrophils (Gr1^+^ F4/80^−^), middle gate represents inflammatory monocytes (Gr1^+^ F4/80^+^), bottom right gate represents resident macrophages (Gr1^−^ F4/80^+^). Data are derived from a single experiment, n = 3 mice/group. Plots for representative animals are shown.

Previous studies have shown that inflammatory monocytes, which are recruited to the peritoneal cavity following i.p. infection [13], are critical for controlling toxoplasmosis in mice [12]. To determine if the altered growth kinetics of ROP5 deficient (RHΔ*ku80*Δ*rop5*) parasites in mice were due to differences in cellular recruitment, we examined the frequency of myeloid cells in the peritoneum at intervals after infection by FACS. Infection with wild type (RH*Δku80*), ROP5 deficient (RH*Δku80Δrop5*) and ROP5 complemented (RH*Δku80Δrop5* Complement) parasites all induced robust recruitment of inflammatory monocytes to the peritoneal cavity by day 3 (Figure 3A, B middle gate). Although the numbers of inflammatory monocytes were similar at day 3, the number of resident monocytes drastically decreased in mice infected with all three strains of *T. gondii* compared to uninfected mice (Figure 3B, Figure S2), a result that is likely due to cell lysis as a consequence of high parasite replication at this time point (Figure 1A). The number of inflammatory monocytes at day 5 also correlated with parasite growth, declining in mice infected with wild type or ROP5 complemented parasites (Figure 3A), while remaining elevated in mice infected with ROP5 deficient parasites. Increased numbers of neutrophils were also observed in infected mice (Figure S2), a phenomenon that has been seen previously in association with high parasite burdens [12]. Collectively these findings indicate that ROP5 deficient parasites initially expand in resident macrophages, but that following recruitment of inflammatory monocytes, the infection is controlled.

### ROP5 and ROP18 mediate survival in inflammatory monocytes by altering the recruitment of IRGs

**Figure 4:**
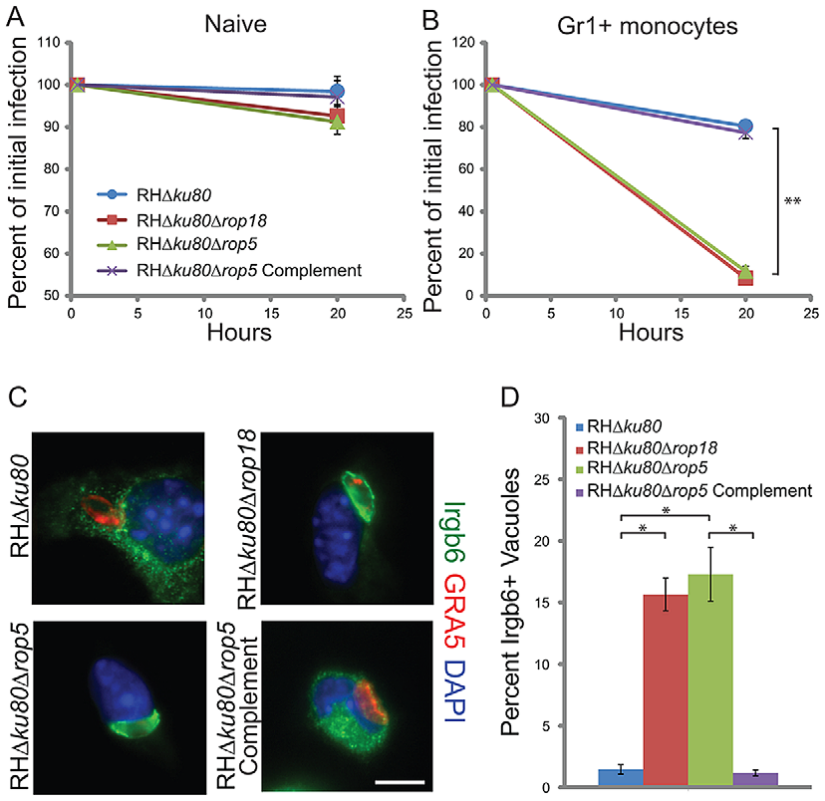
ROP5 and ROP18 are required for avoidance of clearance and IRG recruitment. *In vitro* clearance of parasites in naïve peritoneal macrophages (A) or Gr1^+^ inflammatory monocytes (B) was measured by immunofluorescence microscopy. Cells were stained at 0.5 and 20 h post infection for host cell surface markers (see methods) and the parasite surface marker SAG1. Infection rates at 20 h post infection were normalized to initial infection rates. Means ± S.E.M., n = 3 samples each, from 3 combined experiments. Student's *t* test, **P<0.0005. (C) Immunofluorescence localization of Irgb6 on the parasitophorous vacuole membrane in Gr1^+^ inflammatory monocytes infected for 0.5 h *in vitro*. The vacuole membrane was visualized by staining with mAb Tg 17-113 for GRA5 (secondary: Alexa Fluor 594, red). Irgb6 was visualized with rabbit anti-Irgb6 (secondary: Alexa Fluor 488, green). Scale = 5 microns. (D) Quantification of Irgb6 localization to the vacuolar membrane in Gr1^+^ monocytes. Mean ± SEM, n = 3 samples each, from 3 combined experiments. Student's *t* test, *P<0.005.

Based on the differences in initial growth *in vivo* (Figure 1A), we tested the ability of ROP18 deficient and ROP5 deficient parasites to survive in resident peritoneal macrophages (Gr1^−^ F4/80^+^) vs. inflammatory monocytes (Gr1^+^ F4/80^+^). Naïve macrophages isolated from the peritoneal cavity of normal mice showed limited ability to clear either wild type (RH*Δku80*), ROP18 deficient (RH*Δku80Δrop18*), ROP5 deficient (RH*Δku80Δrop5*) or ROP5 complemented (RH*Δku80Δrop5* Complement) parasites (Figure 4A). In contrast, parasites that were deficient in either ROP18 or ROP5 were efficiently cleared by Gr1^+^ monocytes *in vitro* (Figure 4B), a result that was not accompanied by loss of cells from the monolayer. Survival was completely restored to wild type levels in a strain genetically complemented for ROP5 expression (Figure 4B).

Since ROP18 is known to enhance survival through disrupting IRG recruitment to the parasite containing vacuole [24], we examined the cellular localization of Irgb6 after infection with ROP18 or ROP5 deficient parasites. Although Irgb6 remained diffuse in cells infected with either wild type (RH*Δku80*) and ROP5 complemented (RH*Δku80Δrop5* Complement) parasites, both the ROP18 and ROP5 deficient parasites readily accumulated Irgb6 on the vacuole membrane. The level of Irgb6 recruitment was lower than the extent of clearance in the overnight assay, a result that may be due to kinetic differences (Figure 4C, D). Collectively, these results show that inhibition of recruitment of IRGs by virulent strains of *T. gondii* requires both ROP18 and ROP5.

### ROP5 regulates the catalytic activity of ROP18

**Figure 5:**
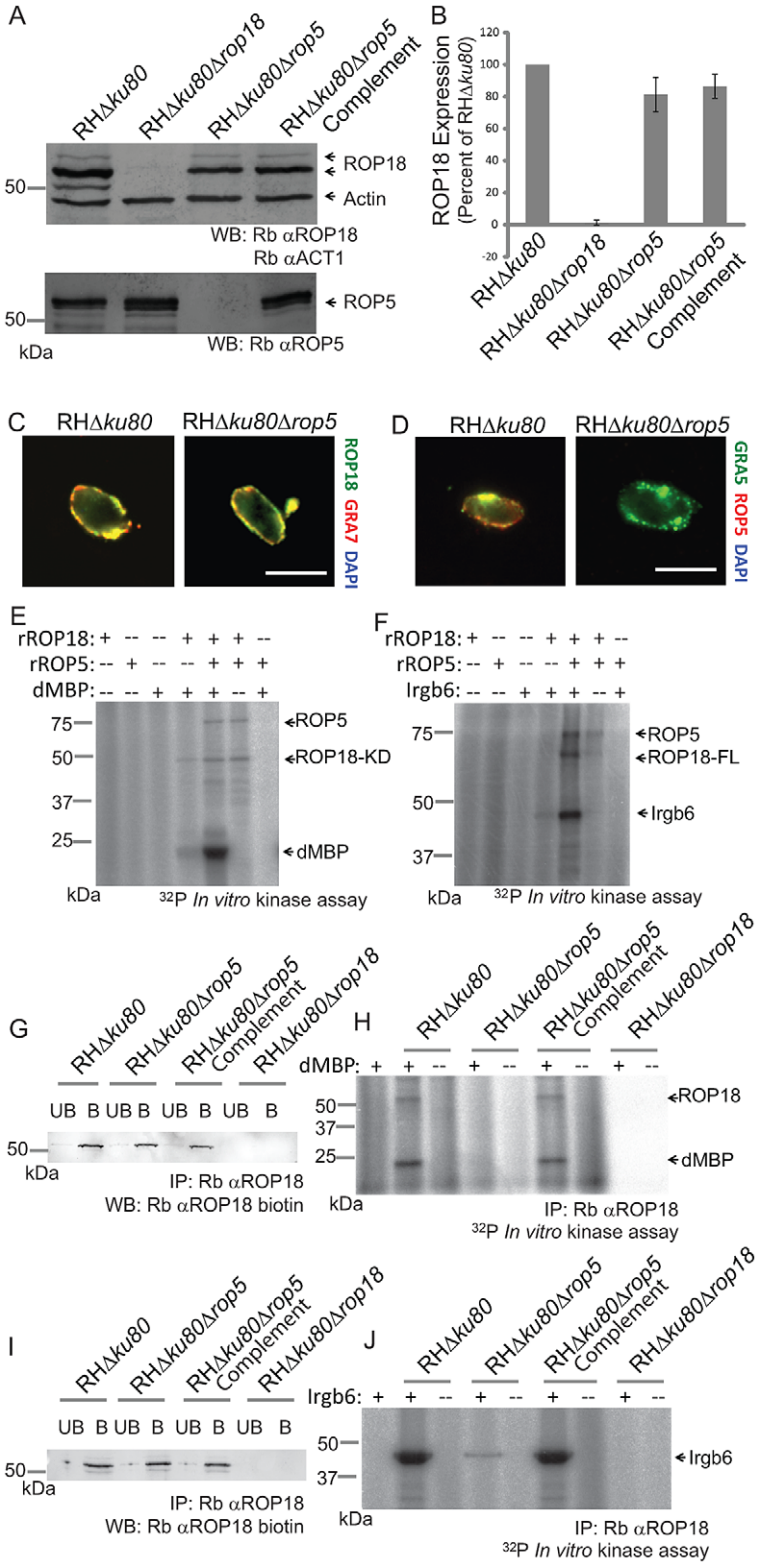
ROP5 regulates the kinase activity of ROP18. (A) Expression of ROP18 and ROP5 detected by western blotting of parasite lysates with rabbit anti-ROP18 (Rb α-ROP18), rabbit anti-ROP5 (Rb αROP5), and rabbit anti-actin (Rb αACT1) as a loading control. Representative of 3 experiments with similar outcomes. (B) Quantification of ROP18 expression by phosphorimager analysis of western blots, normalized for loading by actin staining. Means ± S.E.M. n = 3 experiments. (C) Immunofluorescence localization of ROP18 on the parasitophorous vacuole membrane in wild type (RH*Δku80*) and ROP5 deficient (RH*ΔKu80Δrop5*) parasites infecting HFF cells *in vitro*. ROP18 was localized based on the C-terminal Ty-1 epitope described previously [7] using mAb BB2 (directly conjugated to Alexa Fluor 488, green). The vacuole membrane was stained with polyclonal rabbit anti-GRA7, described previously [64], (secondary: Alexa Fluor 594, red). Scale = 5 microns. (D) Immunofluorescence localization of ROP5 on the parasitophorous vacuole membrane in wild type (RH*Δku80*) and ROP5 deficient (RH*Δku80Δrop5*) parasites infecting HFF cells *in vitro*. The vacuole membrane was labeled with mAb Tg 17-113 for GRA5 (secondary: Alexa Fluor 594, red). ROP5 was labeled with polyclonal Ab for ROP5 (secondary: Alexa Fluor 488, green). Scale = 5 microns. (E) *In vitro* kinase reaction using the kinase domain of ROP18 (ROP18-KD, 100 ng) and the heterologous substrate dMBP ± recombinant ROP5 (rROP5, 200 ng). (F) *In vitro* kinase reaction using full length ROP18 (ROP18-FL, 25 ng) and the natural substrate Irgb6 ± recombinant ROP5 (rROP5, 50 ng). Irgb6 was immunoprecipitated (∼10–20 ng/reaction) from IFN-γ activated RAW cells with polyclonal rabbit anti-Irgb6. (G) Immunoprecipitation of ROP18 (∼5 ng/reaction) from parasite lysates with polyclonal rabbit anti-ROP18 (Rb anti-ROP18). Bound (denoted as B) and unbound (denoted as UB) samples were resolved by SDS-PAGE and blotted for ROP18 (Rb anti-ROP18 biotin). (H) *In vitro* kinase reaction of ROP18 using the heterologous substrate dMBP. ROP18 immunoprecipitations from (G) were incubated with or without substrate in the presence of ^32^P ATP. Reactions were resolved by SDS-PAGE and subjected to phosphorimager analysis. (I) Immunoprecipitation of ROP18 (∼5 ng/reaction) from parasite lysates with rabbit anti-ROP18 as in (G). (J) *In vitro* kinase reaction of ROP18 and a natural substrate Irgb6. ROP18 immunoprecipitations from (I) were incubated with or without substrate in the presence of ^32^P ATP. Irgb6 was immunoprecipitated from IFN-γ activated RAW cells with polyclonal rabbit anti-Irgb6. Reactions in E, F, H, and J were carried out in the presence of ^32^P ATP, resolved by SDS-PAGE and subjected to phosphorimager analysis. E–J are representative of 3 or more experiments with similar outcomes.

Given that ROP18 has previously been shown to be both necessary and sufficient to subvert IRG clearance in murine macrophages infected with *T. gondii* [24], it became important to determine how ROP5 influences this pathway. The dependence of ROP18-dependent functions on ROP5 might result from either altered expression or localization of ROP18. Western blot analysis demonstrated that ROP18 was expressed at near wild-type levels in the absence of ROP5 (Figure 5A,B). Immunofluorescence analysis detected ROP18 properly localized at the vacuolar membrane in both wild type cells (RH*Δku80*) and ROP5 deficient (RH*Δku80Δrop5*) parasites (Figure 5C,D). Collectively, these findings indicate that absence of ROP5 is not responsible for altered expression or localization of ROP18.

Some mammalian pseudokinases have been reported to allosterically regulate active kinases [30], suggesting that ROP5 may regulate ROP18. To test this hypothesis *in vitro*, recombinant ROP18 was used to phosphorylate the artificial substrate dMBP (Figure 5E), or the natural substrate Irgb6 (Figure 5F), in the presence or absence of recombinant ROP5. To determine whether a similar interaction occurs *in vivo*, ROP18 was immunoprecipitated from wild type (RH*Δku80*) parasites or those that were ROP5 deficient (RH*Δku80Δrop5*) (Figure 5G,I) and used to phosphorylate dMBP (Figure 5H) or Irgb6 (Figure 5J) *in vitro*. In the absence of ROP5, both recombinant ROP18 (Figure 5E,F) and endogenous ROP18 (Figure 5H,J), demonstrated a greatly diminished capacity to phosphorylate both dMBP and Irgb6 substrates based on ^32^PO_4_ labeling. Activity of recombinant ROP18 increased ∼12 fold (Figure 5 E,F), while a ∼35 fold increase in endogenous ROP18 activity was observed in the presence of ROP5 (Figure 5J). Restored expression of ROP5 in the complemented clone resulted in phosphorylation of dMBP or Irgb6 by immunoprecipitated ROP18 (Figure 5 H,J). The enhanced activity of ROP18 in the presence of ROP5 did not result from a stable complex between these proteins, as they failed to co-immunprecipitate in lysates of infected, IFN-γ activated cells (Figure S3A). Enhanced activity of ROP18 in the presence of ROP5 also did not result from an interaction between ROP5 and Irgb6 (Figure S3B), nor did the previously demonstrated interaction between ROP18 and Irgb6 [24], require the presence of ROP5 (Figure S3C). Although ROP5 activated ROP18, it failed to demonstrate catalytic activity of its own *in vitro* (Figure 5E,F), consistent with the prediction that it encodes a pseudokinase [27], [28]. Taken together, these results indicate that the catalytic activity of ROP18 is regulated by the predicted pseudokinase ROP5, and that this pathway is required for avoidance of IRG clearance in inflammatory monocytes.

### ROP18 and ROP5 are both required for avoidance of IRG-mediated clearance

Our results indicate that ROP5 and ROP18 are both required for escape from the IRG pathway, consistent with the finding that ROP5 regulates the activity of ROP18. We sought to determine whether the defects in ROP5 and ROP18 could be compensated by defects in the IRG pathway. However, of the two IRG proteins that are shown to be targeted by ROP18, there is presently no knockout available for Irgb6, and the phenotype of Irga6 mutants challenged with *T. gondii* is very modest, especially in cell-autonomous control of parasite survival [33]. Nevertheless, Irgm proteins are known to regulate the proper recruitment of Irga6 and Irgb6 to the vacuole surrounding susceptible strains of *T. gondii* [34], [35]; absence of Irgm1 or Irgm3 alters the targeting and function of Irgb6 and Irga6 and effectively cripples the IRG system. However, the use of mice lacking Irgm1 is complicated by pleomorphic effects of Irgm1-deficiency on T cell development [36] and macrophage motility [37]. In contrast, Irgm3-deficient mice have shown normal immune cell development, yet are highly susceptible to infection by *T. gondii* [17]. Heterologous expression of tagged proteins indicates that Irgm proteins are necessary for proper recruitment of Irga6 and Irgb6 to the vacuole surround susceptible strains of *T. gondii* [35], suggesting the same requirement might be true for endogenous proteins.

**Figure 6:**
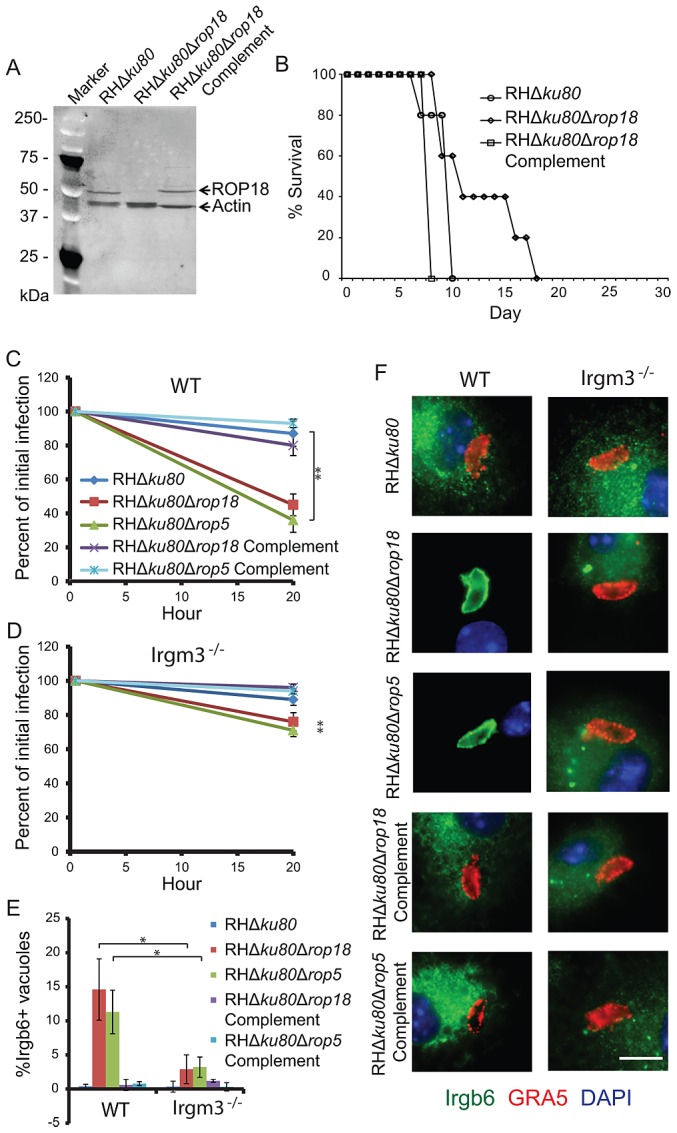
Irgm3 deficiency reverts the IRG recruitment and clearance defects of both Δ*rop18* and Δ*rop5* parasites *in vitro*. (A) Western blot analysis of wild type (RH*Δku80*), ROP18 deficient (RH*Δku80Δrop18*) and ROP18 complemented (RH*Δku80Δrop18*Complement) parasites. Western blot probed with rabbit anti-ROP18 and rabbit anti-actin flowed by goat anti-rabbit conjugated to HRP and detected by ECL. (B) Survival of female outbred CD1 mice challenged with 100 parasites by i.p. inoculation. n = 5 animals per group from a single experiment. *In vitro* clearance of parasites in wild type C57BL/6 (WT) (C) or Irgm3 deficient (Irgm3^−/−^) (D) bone marrow derived macrophages following IFN-γ activation (50 units/mL). Means ± S.D. n = 6 samples from 2 combined experiments. For statistical analysis, parasite survival in Irgm3 deficient cells was compared to survival in wild-type cells. Student's *t* test, **P<0.001. Quantification (E) and immunofluorescence localization (F) of Irgb6 localization to the vacuolar membrane in WT or Irgm3^−/−^ IFN-γ-activated macrophages. (E) Means ± S.D. n = 6 samples from 2 combined experiments. Student's *t* test, *P<0.005. (F) The vacuole membrane was visualized by staining with mAb Tg 17-113 for GRA5 (secondary: Alexa Fluor 594, red). Irgb6 was visualized with rabbit-Irgb6 (secondary: Alexa Fluor 488, green). Scale = 5 microns.

To explore the interaction between ROP5 and ROP18 and the IRG pathway, we examined the recruitment of Irgb6 and parasite clearance in IFN-γ-activated, bone-marrow-derived macrophages derived from wild type C57BL/6 and Irgm3^−/−^ mice. To provide a more complete set of parasite strains for this comparison, we complemented the RH*Δku80Δrop18* mutant described previously [24] by reintroducing a single copy of *ROP18* that restored normal expression and reversed the virulence defect seen in outbred mice (Figure 6A,B, Figure S4, S5). Parasites deficient in ROP18 or ROP5 demonstrated decreased survival in IFN-γ activated wild type macrophages compared to their respective complemented lines or the wild type strain (RH*Δku80*), which is resistant to clearance as previously reported [24] (Figure 6C). The enhanced clearance of ROP5 or ROP18 deficient parasites was largely reverted in IFN-γ activated Irgm3^−/−^ macrophages (Figure 6D). Vacuoles containing both ROP18 deficient (RH*Δku80Δrop18*) and ROP5 deficient (RH*Δku80Δrop5*) parasites showed enhanced Irgb6 accumulation that was restored to normal in the complemented parasite strains in wild type macrophages (Figure 6 E,F). Additionally, the enhanced recruitment of Irgb6 seen in ROP5 or ROP18 deficient mutants was restored to normal in the absence Irgm3 (Figure 6 E,F). Deletion of Irgm3 also affected the abundance and pattern of Irgb6, which tended to aggregate in clusters in the absence of Irgm3 (Figure 6F). These studies reinforce the model that recruitment of Irgb6 is dependent on Irgm3 and establish that ROP18 and ROP5 have indistinguishable phenotypes when it comes to survival in activated macrophages *in vitro*.

**Figure 7:**
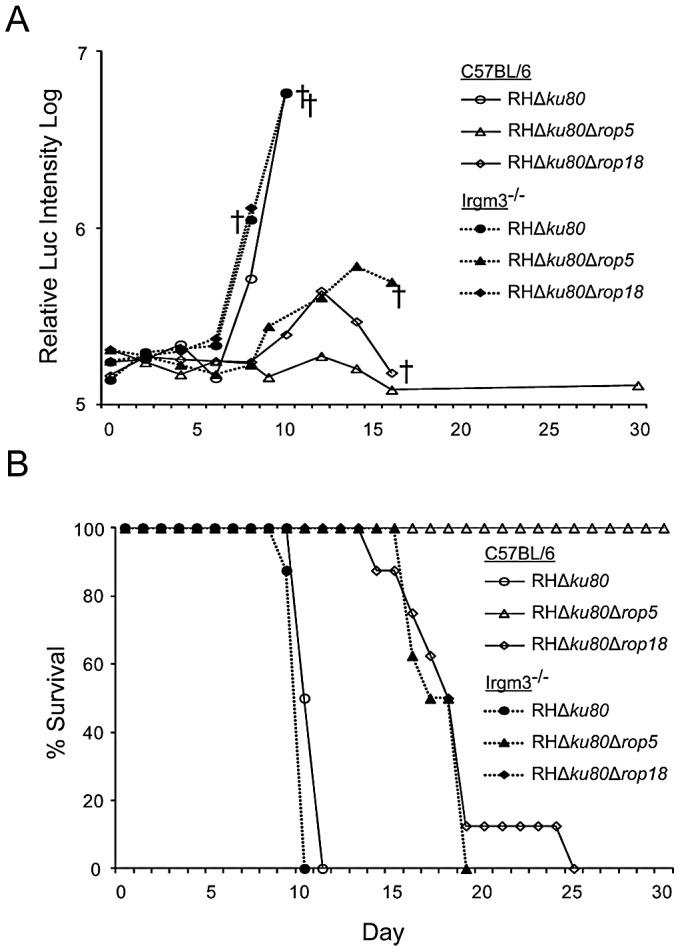
Irgm3 deficient mice revert the phenotypes of Δ*rop18* and Δ*rop5* parasites. (A) Wild type (C57BL/6) (n = 4 per parasite strain) or Irgm3 deficient (Irgm3^−/−^) (n = 4 per parasite strain) mice were infected with 10^2^ luciferase expressing wild type (RHΔ*ku80*), ROP5 deficient (RHΔ*ku80*Δ*rop5*), or ROP18 deficient (RHΔ*ku80*Δ*rop18*) parasites by s.c. injection and imaged on indicated days. † denotes one or more death per group. Representative experiment. (B) Survival curves for mice challenged with *T. gondii* strains as shown. Combination of two experiments (n = 8 animals per group).

To examine survival *in vivo*, wild type and Irgm3^−/−^ mice were challenged with parasites and luciferase activity and survival were recorded. Following s.c. challenge of C57BL/6 mice, wild type (RH*Δku80*) parasites rapidly expanded while ROP5 deficient (RH*Δku80Δrop5*) parasites were controlled as shown by luciferase imaging studies (Figure 7A). Interesting, ROP18 deficient (RH*Δku80Δrop18*) parasites expanded with delayed kinetics in C57BL/6 mice and tissue burdens had begun to recover when animals succumbed to infection (Figure 7A). A similar response was also seen in CD1 outbred mice challenged with ROP18 deficient (RH*Δku80Δrop18*) parasites (Figure S4). In the absence of Irgm3, both wild type (RH*Δku80*) and ROP18 deficient (RH*Δku80Δrop18*) parasites underwent rapid expansion and reached high tissue burdens as reflected by luciferase activity, while ROP5 deficient (RH*Δku80Δrop5*) parasites showed a delayed expansion and reached lower total levels (Figure 7A). The expansion of parasites observed by bioluminescence imaging mirrored survival outcomes. Challenge with wild type strain (RH*Δku80*) parasites led to rapid and complete mortality of both wild type and Irgm3^−/−^ mice (Figure 7B). Wild type mice infected with ROP18 deficient (RH*Δku80Δrop18*) parasites exhibited a delayed death phenotype similar to that seen in outbred mice (Figure 6B), while Irgm3^−/−^ mice succumbed rapidly, similar to wild type parasite infection (Figure 7B). In contrast, ROP5 deficient (RH*Δku80Δrop5*) parasites were completely avirulent in wild type C57BL/6 mice, while they caused 100% mortality in Irgm3^−/−^ mice, albeit with a delay in time to death (Figure 7B). The susceptibility of Irgm3^−/−^ mice infected with ROP5 deficient (RH*Δku80Δrop5*) parasites was more rapid when injected i.p. with a similar time to death as wild type parasites (Figure S6).

## Discussion

Although the pseudokinase ROP5 was previously shown to be essential for acute virulence of *T. gondii* in laboratory mice, the basis for this was initially unclear, especially given the predicted lack of catalytic activity of this protein. Here we demonstrate that ROP5 regulates the activity of ROP18, an active S/T kinase that phosphorylates IRGs, thus blocking their accumulation on the parasite containing vacuole. ROP5 was necessary for the full enzymatic activity of ROP18, although it was not required for stable expression or normal trafficking to the parasite-containing vacuole. Studies using Irgm3 deficient macrophages revealed that the inability of ROP5 and ROP18 deficient parasites to avoid IRG recruitment was fully reverted *in vitro*. Moreover, the attenuation of the ROP deficient mutants was fully reversed in Irgm3 deficient mice. These findings reveal that ROP5 is a multifunctional pseudokinase that regulates acute virulence in *T. gondii* in part by governing the active kinase ROP18, and by affecting additional effectors that are both IFN-γ and Irgm3-dependent.

ROP5 is a member of a polymorphic family of secretory S/T kinases that are highly divergent from human kinases and which have been amplified in the genome of *T. gondii* [8]. Forward genetic mapping revealed that ROP5 is primarily responsible for differences in mouse virulence between highly virulent type I strains and intermediate virulent type II strains [27], and also between type II and avirulent type III strains, although paradoxically the type III ROP5 locus is positively associated with virulence [28]. In all strains, the ROP5 locus encodes a cluster of predicted pseudokinases all of which lack the central conserved Asp residue of the catalytic triad typical of S/T kinases [38]. Our findings demonstrate that the virulence defect in ROP5 deficient parasites is completely reversed in mice lacking Ifngr1^−/−^ or Rag1^−/−^, indicating that ROP5 mediates escape from IFN-γ-dependent effector mechanisms. Alternative models, such as ROP5 deficient parasites being auxotrophic for nutrients that may be limiting *in vivo*, or ROP5 being a global suppressor of immune responses, are not supported.

Previous studies have shown that IFN-γR1 is required for control of *T. gondii* in both hematopoietic and non-hematopoietic cells [32], and both compartments likely contribute to IRG-mediated clearance, which in the mouse provides one of the most effective means of control [21], [22]. At the level of survival in macrophages *in vitro*, ROP5 and ROP18 were both required for avoidance for recruitment of IRGs and clearance. In previous studies we have shown that ROP18 deficient parasites exhibit normal survival in naive macrophages, but are restricted in IFN-γ activated peritoneal macrophages and that survival correlates with avoidance of Irgb6 recruitment [24]. Here we extend these findings to show that ROP18 or ROP5 deficient parasites show enhanced Irgb6 recruitment and clearance in Gr1^+^ monocytes, and in bone marrow derived macrophages activated *in vitro* with IFN-γ. In separate studies, others have shown that ROP18 or ROP5 deficient parasites also fail to block recruitment of Irga6 and Irgb6 in IFN-γ-activated MEFs [39]. In the present study, the increased susceptibility of ROP18 and ROP5 deficient parasites to clearance by IFN-γ-activated macrophages was completely dependent on Irgm3, a regulatory protein required for homeostasis of IRGs. Moreover, deficiency in Irgm3 reverted the phenotype of both the Δ*rop18* and the Δ*rop5* mutants *in vivo*.

Previous genetic analyses of acute virulence in type I strains of *T. gondii* indicated that ROP5 and ROP18 interact to control virulence [7], [27]. We now demonstrate that the basis for this relationship is that ROP5 controls the kinase activity of ROP18, thus affecting its ability to phosphorylate substrates. ROP5 activation of ROP18 activity contributes to avoidance of IRG recruitment in IFN-γ activated macrophages, hence promoting survival. ROP18 actively phosphorylates a number of IRG proteins in a common motif in switch region I [24], thereby affecting GTPase activity, and oligomerization [25]. Following phosphorylation, IRG proteins are unable to load onto the parasite containing vacuole, thus blocking this interferon-mediated clearance pathway [40]. Previous studies have indicated that Irgm3 is recruited to the PVM [19], [41], and it contains the conserved motif [24], and therefore is a potential substrate of ROP18. Additionally, the activity of ROP18 in phosphorylating ATF6β [42], is likely to also dependent on ROP5. ATF6β has been proposed to affect a later step in resistance mediated through dendritic cell activation of T-cells, a process likely important in adaptive immunity [42]. At an earlier stage, ROP18 is essential for controlling avoidance of the IRG pathway, a process that participates primarily in innate immunity [43]. Collectively, these two pathways likely control the ROP5-dependent activities of ROP18 in mediating virulence.

Our findings are consistent with a model whereby ROP5 acts as an allosteric regulator of ROP18. ROP5 was required for full catalytic activity of ROP18 using endogenous enzyme immunoprecipitated from cells and *in vitro* testing against the heterologous substrate dMBP or the natural substrate Irgb6. ROP5 also directly activated the kinase activity of recombinant ROP18 *in vitro* against both dMBP and Irgb6. These results are reminiscent of recent reports of a role for pseudokinases in mammalian cells regulating their active partners [30], [44]. For example, the pseudokinase STRADα regulates LKB1, a S/T protein kinase that regulates AMP activated kinase and acts as a tumor suppressor [45]. Although catalytically inactive, STRADα adopts a closed conformation typical of an active kinase and together with the adaptor MO25α promotes the active conformation of LKB1 [46], [47]. Unlike the situation with LKB and STRADα, the activation of ROP18 occurs despite there not being a strong interaction with ROP5, which does not coIP from cell lysates [48], and present report. However, the failure to observe a stable complex under these conditions does not preclude ROP5 and ROP18 from interacting at a lower affinity, or in a complex that depends on local interactions on the PVM. It is conceivable that transient binding of ROP5 to ROP18 facilitates auto-catalytic activation, which results from phosphorylation in helical extensions of the N-lobe of the kinase domain [10]. In separate studies, using a more sensitive approach based on tandem-affinity purification (TAP) [49] of ROP5, ROP18 was one of the major components to copurify in a complex with ROP5, and this was validated by reciprocal TAP-tagging of ROP18 (Etheridge, Sibley unpublished). Moreover, we observed that ROP5 is found in a complex with other ROP kinases, suggesting it may activate other kinases similar to ROP18 (Etheridge, Sibley unpublished). Although the precise mechanism of regulation is yet unclear, our data indicate that ROP5 is an allosteric activator of ROP18, thus establishing a new role for pseudokinases in controlling pathogen virulence factors.

Our findings differ from a recent report that also examined the interaction of ROP5 and ROP18 [48]. This prior study reported that co-expression of a cosmid contain the locus of *ROP5* from the type I strain was not able to enhance the activity of ROP18 from a type II strain, when co-transfected into a recombinant strain called S22 [48]. Interpretation of this experiment is complicated by the fact that S22 is the product of recombination between types II and III and it also contains the type II *ROP5* locus, which has previously been associated with avirulence [27]. Either due to this complex mixture of ROP5 alleles, or another undefined locus, this strain may harbor an epistatic activity that suppresses activation of ROP18. In contrast we demonstrate that the major allele of ROP5 from the type I strain activates the kinase activity of ROP18 from a type I strain *in vivo*, using isogenic strains, and *in vitro* using purified recombinant protein to phosphorylate both heterologous and endogenous substrates. This later result was also observed using GST-Irga6 as a substrate *in vitro* [39], confirming the ability of ROP5_I_ to enhance the activity of ROP18_I_.

Two recent studies also reported that ROP5 binds directly to some IRGs, notably Irga6, affecting its oligomerization and GTPase activity *in vitro* [39], [48]. This result suggests that ROP5 may also inhibit oligomerization *in vivo*, thus decreasing IRG accumulation on the parasite-containing vacuole. However, this activity alone is unlikely to be sufficient for parasite survival, given the inability of type III strains to avoid IRG recruitment despite expressing the same complement of *ROP5* alleles as seen in type I strains [27]. Additionally, although ROP5 binds reasonably well to Irga6, it binds less efficiently to other IRGs such as Irgb6 ([39] and present study). As such, the ability of ROP5 to directly activate the kinase activity of ROP18 may be more important for targets such as Irgb6. Collectively, the binding of ROP5 to IRGs and inhibition of oligomerization is expected to work cooperatively with its ability to enhance the catalytic activity of ROP18, thus disrupting IRG function.

The IRG pathway has been described as a major immunity mechanism in the murine system due to the expansion of this family of innate immune effectors in the rodent lineage [20]. The nearly exclusive expression of IRGs in the murine system has led some to question its relevance to human infection. However, this view overlooks the obvious importance of rodents in the natural transmission of toxoplasmosis, which is a zoonotic disease that humans acquire from infected food animals and cats, although not directly from rodents. Additionally, there are several reasons to believe IRGs are also directly relevant to humans. Humans express only two IRG family members: IRGC, which is testis specific and unlikely to be involved in immunity, and IRGM, which is truncated [20]. Despite likely not being a functional GTPase, IRGM has been implicated in autophagy-mediated control of *Mycobacterium tuberculosis* [50] and *Salmonella typhimurium* [51]. Additionally, both humans and rodents express a second family of related GTPases called guanylate binding proteins (GBPs), which are also strongly upregulated following treatment with IFN-γ [52]. GBPs have recently been shown to be required for the control of *Listeria monocytogenes* and *M. tuberculosis* in the mouse [53]. GBPs are also recruited to vacuoles containing *T. gondii* in a strain-dependent manner [52], [54], suggesting a role in pathogen control. Direct evidence for such a role was recently provided by a study reporting deletion of a locus on chromosome 3 in the mouse, encoding 5 GBPs, impairs immunity to challenge with a type II strain of *T. gondii* [55]. In separate studies, we have shown that these effects are partially dependent on Gbp1 and that recruitment of Gbp1 to the PVM is mediated in a ROP5 and ROP18-dependent manner (Selleck, Sibley submitted). Homeostasis of GBPs requires Irgm proteins in the murine system [56], hence the dramatic reversal of the ROP5 deficient parasites in Irgm3^−/−^ mice may be due to defects in both the IRG and GBP systems. Further studies will be needed to determine the relationship between these different IFN-γ induced systems and to define the role of ROP kinases and IRG-dependent immunity mechanisms in control of human infection.

Previous genetic crosses have implicated only a few loci in controlling acute virulence in the mouse model [6], [22], [28], [57]. Notably, only type I parasites are efficient at blocking IRG clearance [14], [23] and they have a combination of a type I allele at ROP18 and a type I allele at ROP5, the latter of which is also expressed by type III parasites [7], [27]. Consistent with their extremely low level of ROP18 expression, type III parasites are avirulent, a phenotype that is fully reverted with transgenic expression of type I [7] or type II [6] ROP18. Although type II strain parasites have a functional ROP18, they have a type II ROP5 locus that is associated with avirulence. Although we have not tested the ability of type II ROP5 to regulate the activity of the type II allele of ROP18, genetic studies indicate that this interaction is not sufficient to promote full virulence [28], [57], nor is it sufficient to mediate avoidance of IRG recruitment and clearance [14], [23].

The more severe defect of ROP5 deficient parasites *vs.* ROP18 deficient parasites in wild type mice, suggest that in addition to regulating ROP18, it has other functions, perhaps serving as scaffold for regulating other important kinases in *T. gondii*. ROP kinases are highly polymorphic and have expanded in the *T. gondii* genome under strong selective pressure [8]. Similarly, the IRG pathway is highly amplified in rodents where it plays a major role in resistance to intracellular pathogens such as *T. gondii*. Placing the ROP5 pseudokinase at the center of this pathway may be an evolutionary strategy to divert attention from the active kinases, in which diversity is constrained to preserve catalytic activity.

## Materials and Methods

### Ethics statement

All animal experiments were conducted according to the U.S.A. Public Health Service Policy on Humane Care and Use of Laboratory Animals. Animals were maintained in an AAALAC-approved facility and all protocols were approved by the Institutional Animal Care and Use Committee (School of Medicine, Washington University in St. Louis).

### Mouse strains and infectivity studies

CD-1 and C57BL/6 mice were purchased from Charles River Laboratories. Ifnγr1^−/−^, Rag1^−/−^, and Nos2^−/−^ mice,, all on a C57/BL6 background were obtained from Dr. Herbert Virgin (Washington University). Mice deficient in the superoxide-generating NADPH-oxidase gp91^phox^ subunit (NOX2), referred to as X-CGD mice, on a C57BL/6 background [58] were obtained from Dr. Mary C. Dinauer (Washington University). Irgm3^−/−^ mice [17] from Dr. Greg Taylor (Duke University) were bred locally at Washington University. Age and sex- matched mice were challenged by i.p. or s.c. injection with parasites and survival followed for 30 days post injection, as described previously [27].

### *In vivo* imaging and cytokine measurements

Mice inoculated with luciferase expressing parasites were weighed at intervals after infection and imaged by bioluminescence as described previously [59]. For cytokine measurements, blood was obtained from the saphenous vein. Sera were obtained using microtainer serum separators (BD Bioscience) by centrifugation at 3,000 *g* and stored at −20°C. IL-12p40 was measured using a mouse OptEIA ELISA kit (BD Biosciences). The other cytokines were quantified using the Cytometrix Bead Array Mouse Inflammation Kit (BD Biosciences), detected using a FACS Canto II flow cytometer (BD Biosciences), and analyzed using FCAP ARRAY (Soft Flow, Inc.).

### Parasite and cell culture

Fully virulent, type I RH strain parasites that are deficient in Ku80 (RH*Δku80*), attenuated Δ*rop18* (RH*Δku80Δrop18*), avirulent Δ*rop5* (RH*Δku80Δrop5*), and virulent ROP5 complemented (RH*Δku80Δrop5*Complement) parasite strains were described previously [24], [27]. Generation of the ROP18 complement (RH*Δku80Δrop18*Complement) is described in Figure S4. Parasites were serially passaged in human foreskin fibroblasts (HFF) monolayers, as described previously [24]. Cultures were negative for mycoplasma contamination using the e-Myco plus mycoplasma PCR detection kit (Boca Scientific). Peritoneal macrophages harvested from naïve CD1 mice, bone marrow derived macrophages, and RAW 264.7 cells (American Type Culture Collection (ATCC)) were cultured as described previously [24]. Cells were activated by treatment overnight with either 10 or 50 units/mL murine IFN-γ (R&D Systems) and 0.1 ng/mL LPS from *E. coli* O55:B5 (Sigma-Aldrich). Gr1^+^ monocytes were harvested at day 4 from the peritoneal cavity of mice that have been primed 4 days previously by inoculation with 200 CTG strain parasites, as described previously [24]. Macrophages were characterized by expression of the cell surface markers using mAb RB6-8C5 against Gr1 that was directly conjugated to Alexa Fluor 594 and/or mAb HB-198 (ATCC) against F4/80 that was directly conjugated to Alexa Fluor 488, using commercially available coupling kits (Invitrogen).

### Generation of luciferase expressing parasites

The pDestR4R3-UPRTKO-Clickluc plasmid was constructed with the MultiSite Gateway 3-fragment pDest-R4R3 system (Invitrogen). Flanking fragments (1 kb 5′ pDONR-P41r and 3′ pDONR-P2rP3) for the *T. gondii* uracil phosphoribosyl transferase gene (*UPRT)* gene were amplified from RH strain lysate with iProof High Fidelity DNA polymerase (Bio-Rad). The middle fragment (pDONR-P1P2) contained the Click Beetle luciferase (*Clickluc*) gene driven by the dihydrofolate reductase (*DHFR*) promoter as described [60] (Table S1). RHΔ*ku80*, RH Δ*ku80*Δ*rop18*, or RHΔ*ku80*Δ*rop5* parasites (1), were electroporated with pDestR4R3-UPRTKO-Clickluc linearized with BsiWI, selected with fluorodeoxyuridine (FUDR)(1×10^−5^ M), and luciferase positive single cell clones were identified by positive bioluminescence.

### Generation of ROP18 complemented parasites

A 5-fragment Gateway clone was generated to integrate the *ROP18* gene under control of the *IMC1* promoter into the *UPRT* locus (Figure S5). The targeting construct was amplified by PCR as described previously [61], and electroporated into RHΔ*ku80*Δ*rop18* strain parasites, stable transformant clones isolated and verified by immunofluorescence staining and western blotting.

### Cell recruitment assay

Recruitment of cells to the peritoneal cavity was analyzed by FACS, using protocols described previously [59]. In brief, mice were sacrificed at intervals after infection, peritoneal cells were isolated, and stained and analyzed by FACS. Cells were incubated at 4°C in Fc block (clone 2.4G2, BD Bioscience) in MACS buffer (PBS, 0.5% BSA, 2 mM EDTA, pH 7.2) and negative cells excluded by staining with AmCyan-Aqua Fixable Dead Cell Stain (Invitrogen). Labeled antibodies V450 anti-Gr1 (RB6-8C5, BD Bioscience), APC-anti-F4/80 (Invitrogen), PE-Cy7 anti-CD11b (M1/70, BD Bioscience), and APC-eFluor 780 anti-B220 (RA3-6B2, eBioscience) were incubated for 15 min at room-temp, washed and re-suspended in MACS buffer. Samples were detected using a FACS Canto II flow cytometer (BD Biosciences), and analyzed with FlowJo software (Tree Star, Inc). Absolute cell numbers were calculated using the total cell count multiplied successively by the percentages for the appropriate gates obtained through flow cytometry. A total of 100,000 cells were analyzed for each sample.

### Immunofluorescence microscopy

Infected HFF or macrophage monolayers cultured on coverslips were fixed in 4% formaldehyde and permeablized in 0.05% Triton X-100 in PBS or 0.05% saponin for 10 min. Samples were blocked with 10% FBS, incubated with primary antibodies for ∼20 min, washed 3 times with PBS, and incubated with species-specific secondary antibodies conjugated to Alexa Fluors (Invitrogen) for ∼20 min. Samples were rinsed in PBS, mounted in ProLong Gold with DAPI (Invitrogen) and examined with a Zeiss Axioskop 2 MOT Plus microscope (Carl Zeiss, Inc.). Images were acquired with an AxioCam MRm camera (Carl Zeiss, Inc.) and processed with Photoshop CS4.

### *In vitro* macrophage clearance assays

Macrophage monolayers were challenged with parasites that were propagated in HFF cells as described above. Clearance was assessed by comparing the percentage of cells infected following a 30 min infection pulse vs. those remaining after 20 h, as described previously [24]. The numbers of infected cells were determined by counting of 10 fields using a 40× objective lens from 3 replicates per condition. Two or three replicate experiments were performed for each assay.

### Accumulation of IRGs to the parasite containing vacuole

Gr1^+^ monocytes or IFN-γ treated bone marrow derived macrophages were challenged with parasites for 30 min, fixed in formalin buffered saline, and processed for immunofluorescence, as described previously [24]. *T. gondii* containing vacuoles were stained with mAb Tg17–113, which recognizes dense granule protein 5 (GRA5) [62]. Irgb6 was localized with rabbit anti-Irgb6 [34], and the numbers of positive vacuoles were determined by counting of 10 fields using a 40× objective lens from 3 replicates per condition. Two or three replicate experiments were performed for each assay.

### Western blot analysis

Parasite lysates were resuspended in denaturing Laemmli sample buffer, resolved in 10% acrylamide gels, transferred to nitrocellulose and probed with rabbit anti-ROP18 [24], rabbit anti-ROP5 [27], or rabbit anti-actin [63]. Blots were washed, incubated with goat anti-rabbit IgG conjugated to HRP (Jackson ImmunoResearch), and detected using the ECL Plus western blotting system (GE Healthcare) and FLA5000 phosphorimager analysis (Fuji Life Sciences).

### Immunoprecipitation

Immunoprecipitations were performed as described previously [24]. In brief, cells were lysed 1% NP-40, 50 mM Tris–HCl, 150 mM NaCl, pH 8.8 plus protease inhibitors, centrifuged at 1,000 *g* 4°C, and pre-cleared by incubation with protein G sepharose (Pierce Biotechnology Inc.) for 1 h at 4°C. ROP18 was immunoprecipitated using either the mAb BB2 against the Ty-1 tag, or polyclonal rabbit anti-ROP18. Irgb6 was immunoprecipitated from IFN-γ activated bone marrow derived macrophages with rabbit anti-Irgb6, and purity was confirmed by MS/MS, as described previously [24]. Protein G sepharose was charged with antibodies for 1 h at room temperature, washed with PBS, incubated with cell lysates overnight at 4°C, followed by washing with PBS. The efficiency of ROP18 precipitation was assessed by probing with rabbit anti-ROP18 that was directly conjugated to NHS-biotin (Pierce, Thermo Scientific), washed in PBS, incubated with streptavidin conjugated to HRP, and detected as described above for western blotting.

### Production of recombinant proteins

ROP18-kinase domain (ROP18-KD) was expressed and purified as described previously [10]. Genomic DNA from the type I RH strain of *T. gondii* was used to amplify the genes encoding full length ROP18 (ROP18-FL) (starting from Glu83 based on the second ATG of GenBank protein CAJ27113) or ROP5 (starting from Val25 of GenBank protein AAZ73240.1) using iProof high-fidelity polymerase (Bio-Rad) and primers listed in Table S1. Amplicons were cloned into pGEX-6P-1 using primers that introduced a C-terminal His_6_ tag. ROP18-FL was expressed in BL21 (DE3)-V2R-pACYC LamP, as described previously [10]. ROP5-FL was expressed in Rosetta (DE3)pLysS (Novagen). Cells were induced with 1 mM IPTG, grown overnight at 15°C, and soluble proteins were purified using Glutathione Sepharose 4B (GE Healthcare) according to the manufacturer's recommendations. Protein purity and concentration were assessed by SDS-PAGE and SYPRO Ruby staining.

### *In vitro* ^32^P kinase assays

Kinase activity of recombinant or immunoprecipitated ROP18 was tested on the heterologous substrate dephosphorylated myelin basic protein (dMBP) (Millipore) (0.5 µg/reaction), or separately on Irgb6 that was immunoprecipitated from IFN-γ activated RAW cells using rabbit anti-Irgb6. Kinase reactions were conducted in 25 mM Tris–HCl pH 7.5, 15 mM MgCl_2_ and 2 mM MnCl_2_. containing 10 µCi of ^32^P γ-ATP (specific activity: 3,000 Ci/mmol) (Perkin Elmer, Inc) in addition to 33 µM unlabelled ATP (Sigma-Aldrich). Reactions were allowed to proceed at 30°C for 30 min, samples were heated to 95°C in Laemmli sample buffer, resolved on 12% or 10% SDS–PAGE gels, dried, and imaged using a FLA5000 phosphorimager.

### Statistics

Statistical calculations were performed in Excel. Student's *t* tests were performed under the assumption of equal variance and using a two-tailed test,, where *P*≤0.05 was considered significant.

### Host expression microarray analysis

Confluent HFF monolayers grown in T75 flasks were infected with 12×10^6^ wild type (RHΔ*ku80*) or ROP5 deficient (RHΔ*ku80*Δ*rop5*) parasites (MOI of 4), or mock infected and allowed to grow for 24 hours. Cells were washed in PBS lacking divalent cations, removed by trypsinization, washed in DMEM containing 10% FBS, pelleted by centrifugation at 400 *g* for 10 min, and the pellets stored at −80°C. To extract RNA, pellets were thawed and processed with the Qiagen RNeasy kit supplemented with β-mercaptoethanol and DNase I treatment (Qiagen). Total RNA was processed and labeled into cRNA using the Ambion Message Amp Premier (Ambion) according to the manufacturer's protocol using 500 ng starting RNA. A total of 10 µg cRNA was hybridized to the HG-U113A_2 Affymetrix Human Genome Array (Affymetrix) using standard manufacturer's hybridization and scanning protocols. Data was processed using GeneSpring 7.2 (Agilent Technologies) with the following normalizations: Robust Multi-array Average (RMA), Data transformation: Set measurements less than 0.01 to 0.01, Per Chip and Per Gene: Median Polishing. There was only one gene >2.5 fold different between RHΔ*ku80* and RHΔ*ku80*Δ*rop5* infected host samples, a serine protease inhibitor - BC005224 (Genbank id for the probe set specific for this gene; http://www.ncbi.nlm.nih.gov/nuccore/BC005224) and the variance in the RHΔ*ku80*Δ*rop5* data for this gene resulted in the difference being non-significant. Data was submitted to NCBI GEO record GSE32104.

## Supporting Information

Figure S1 **Luciferase imaging of infected mice.** CD-1 mice infected with either 10^6^ (A) or 10^3^ (B) luciferase expressing wild type (RHΔ*ku80*) or ROP5 deficient (RHΔ*ku80*Δ*rop5*) parasites, Day 0 through Day 8. Mice were injected i.p. with D-luciferin (Biosynth AG) at 150 mg/kg, anesthetized with 2% isoflurane for 5 min and imaged with a Xenogen IVIS 200 Imager and processed using Xenogen Living Image software (Caliper Life Sciences). Quantification of these images was used to graph data in Figure 1A.
(TIF)

Figure S2 **FACS analyses of cell populations in the peritoneum of challenged mice.** (A) CD-1 mice were infected with 10^3^ wild type (RHΔ*ku80*), ROP5 deficient (RHΔ*ku80*Δ*rop5*), or ROP5 complemented (RHΔ*ku80*Δ*rop5*Complement) parasites and the number of Gr1^+^ F4/80^−^ cells (A, neutrophils) and Gr1^−^ F4/80^+^ cells (B, resident macrophages) was determined by cell surface staining and FACS. Mean ± S.E.M., n = 3 animals per group. Representative experiment. Data in (A) and (B) were derived from the same cell populations as those in Figure 3A.
(TIF)

Figure S3 **Irgb6-ROP18 protein interaction is present in the absence of ROP5.** RAW cells were activated for 24 hr with 10 U/mL IFN-γ and 0.1 ng/mL LPS prior to infection for 30 min with indicated strains. (A) Immunoprecipitation of ROP5 from infected cell lysates with polyclonal rabbit anti-ROP5 (Rb αROP5). Unbound (UB) and bound (B) fractions were resolved by SDS-PAGE and blotted for ROP18 (Rb αROP18 biotin). ROP18 is denoted by the double arrows representing both full length and proteolytically processed protein. A third lower molecular weight nonspecific band present in all bound fractions likely represents heavy chain antibody. (B) Immunoprecipitation of ROP5 from infected cell lysates with polyclonal rabbit anti-ROP5 (Rb αROP5). Unbound (UB) and bound (B) fractions were resolved by SDS-PAGE and blotted with goat anti-Irgb6 (Gt αIrgb6, Santa Cruz A-20, denoted by arrow). (C) Immunoprecipitation of Irgb6 from infected cell lysates with polyclonal rabbit anti-Irgb6 (Rb αIrgb6). Bound (B) fractions were resolved by SDS-PAGE and blotted for ROP18 (Rb αROP18 biotin, denoted by double arrows).
(TIF)

Figure S4 **Luciferase imaging of RHΔ** ***ku80*** **Δ** ***rop18*** **infected CD-1 mice.** CD-1 mice were i.p. injected with either 10^3^ luciferase expressing wild type (RHΔ*ku80*) or ROP18 deficient (RHΔ*ku80*Δ*rop18*) parasites and imaged on indicated days. † denotes one or more deaths. Mean values shown per group (n = 5). Representative experiment. (B) Survival curves for mice challenged in A. Representative experiment, n = 5 mice per group. Although luciferase signals returned to background levels in some mice before they expired, the presence of parasites in these mice could be detected with longer exposure, suggesting the mice eventually succumbed from low burden chronic infections.
(TIF)

Figure S5 **Gateway cloning construction of ROP18 complementation plasmid.** The Gateway Cloning Technology (Invitrogen) is currently designed to allow for cloning of 1 to 4 separate fragments using site-specific recombinases (http://www.invitrogen.com/site/us/en/home/Products-and-Services/Applications/Cloning/Gateway-Cloning.html). We utilized currently available Gateway plasmids to construct a 5-fragment cloning strategy. By combining two 3-fragment MultiSite Gateway systems, pDEST-R1R2 and pDEST-R4-R3, we generated a 5-fragment plasmid that was used to complement *ROP18*. The middlemost cassette was first cloned into pDEST-R1R2 and used as the center fragment (pDONR-P1P2) in the second round of cloning into pDEST-R4R3. Although there are internal attB4 and attB3 sites in this center fragment, they do not hinder the ability to obtain a correct entry clone after the BP reaction with pDONR-P1P2 or an expression clone after the LR reaction with the 3-fragment pDEST-R4R3 system. To create the ROP18 complementation plasmid, we combined three fragments contained in separate plasmids: a genomic region 1,000 bp upstream of the *IMC1* gene, assumed to contain its promoter, (*i.e.* TGME49_031630) (pDONR-P1P4); the *ROP18* gene with a 3′ Ty tag (pDONR-P4rP3r); and the *DHFR* 3′ UTR (pDONR-P3P2) into the pDEST-R1R2 system. The *IMC1* promoter and *ROP18* gene were amplified from RH strain lysate using iProof High Fidelity DNA polymerase (Bio-Rad). The 3-fragment pDEST-R1R2 plasmid was used to clone the IMC1p-ROP18Ty-DHFR 3′ UTR fragment into pDONR-P1P2. This plasmid was then combined with the plasmids containing flanking regions of the UPRT gene used to construct pDestR4R3-UPRTKO-Clickluc (see Material and Methods) into the pDEST-R4R3 system to create the final 5-fragment plasmid referred to as pDEST-R4R3 (5-Frag)-UPRTKO-IMC1p-ROP18Ty-DHFR3′. Primers used for the various plasmids are found in Table S1. The fragment containing the UPRT 5′ KO-IMC1p-ROP18Ty-DHFR 3′ UTR- UPRT 3′ KO target sequence was PCR amplified, as described previously [61], from pDEST-R4R3(5-Frag)-UPRTKO-IMC1p-ROP18Ty-DHFR3′ with iProof High Fidelity DNA polymerase (Bio-Rad), purified using the QIAquick PCR Purification Kit (Qiagen), and resuspended in cytomix. The purified template was electroporated into RHΔ*ku80*Δ*rop18* parasites and selected with fluorodeoxyuridine (FUDR)(1×10^−5^ M). Stable parasites were cloned and assessed for Ty expression by immunofluorescence assay using anti-Ty Ab conjugated to Alexa Fluor 488 (Invitrogen). ROP18 was assessed by Western blot using primary rabbit anti-ROP18 or rabbit anti-Actin detected with secondary anti-rabbit-HRP, as described previously [27].
(TIF)

Figure S6 **Luciferase imaging of i.p. injected Irgm3^−/−^ mice.** (A) Wild type (C57BL/6) (n = 4 per parasite strain) or Irgm3 deficient (Irgm3^−/−^) (n = 4 per parasite strain) mice were infected with 10^2^ luciferase expressing wild type (RHΔ*ku80*), ROP5 deficient (RHΔ*ku80*Δ*rop5*), or ROP18 deficient (RHΔ*ku80*Δ*rop18*) parasites by i.p. injection and imaged on indicated days. † denotes one or more death per group. Representative experiment. (B) Survival curves for mice challenged with *T. gondii* strains as shown. Combination of two experiments (n = 8 animals per group).
(TIF)

Table S1 **Primers used in this study.**
(PDF)

## References

[1] Dubey JP (2010) Toxoplasmosis of animals and humans. Boca Raton: CRC Press. 313 p.

[2] Howe DK, Sibley LD (1995) *Toxoplasma gondii* comprises three clonal lineages: correlation of parasite genotype with human disease. J Infect Dis 172: 1561–1566. 7594717 10.1093/infdis/172.6.1561

[3] Khan A, Dubey JP, Su C, Ajioka JW, Rosenthal BM, et al (2011) Genetic analyses of atypical *Toxoplasma gondii* strains reveals a fourth clonal lineage in North America. Int J Parasitol 41: 645–655. 21320505 10.1016/j.ijpara.2011.01.005 PMC3081397

[4] Khan A, Taylor S, Su C, Mackey AJ, Boyle J, et al (2005) Composite genome map and recombination parameters derived from three archetypal lineages of *Toxoplasma gondii* . Nuc Acids Res 33: 2980–2992. 10.1093/nar/gki604 PMC1137028 15911631

[5] Su C, Howe DK, Dubey JP, Ajioka JW, Sibley LD (2002) Identification of quantitative trait loci controlling acute virulence in *Toxoplasma gondii* . Proc Natl Acad Sci U S A 99: 10753–10758. 12149482 10.1073/pnas.172117099 PMC125035

[6] Saeij JPJ, Boyle JP, Coller S, Taylor S, Sibley LD, et al (2006) Polymorphic secreted kinases are key virulence factors in toxoplasmosis. Science 314: 1780–1783. 17170306 10.1126/science.1133690 PMC2646183

[7] Taylor S, Barragan A, Su C, Fux B, Fentress SJ, et al (2006) A secreted serine-threonine kinase determines virulence in the eukaryotic pathogen *Toxoplasma gondii* . Science 314: 1776–1780. 17170305 10.1126/science.1133643

[8] Peixoto L, Chen F, Harb OS, Davis PH, Beiting DP, et al (2010) Integrative genomics approaches highlight a family of parasite-specific kinases that regulate host responses. Cell Host Microbe 8: 208–218. 20709297 10.1016/j.chom.2010.07.004 PMC2963626

[9] Labesse G, Gelin M, Bessin Y, Lebrun M, Papoin J, et al (2009) ROP2 from *Toxoplasma gondii*: a virulence factor with a protein-kinase fold and no enzymatic activity. Structure 17: 139–146. 19141290 10.1016/j.str.2008.11.005

[10] Qiu W, Wernimont A, Tang K, Taylor S, Lunin V, et al (2008) Novel structural and regulatory features of rhoptry secretory kinases in *Toxoplasma gondii* . EMBO J 28: 969–979. 10.1038/emboj.2009.24 PMC2670854 19197235

[11] Yap GS, Sher A (1999) Cell-mediated immunity to *Toxoplasma gondii*: initiation, regulation and effector function. Immunobiol 201: 240–247. 10.1016/S0171-2985(99)80064-3 10631573

[12] Dunay IR, DaMatta RA, Fux B, Presti R, Greco A, et al (2008) Gr1^+^ inflammatory monocytes are required for mucosal resistance to the pathogen *Toxoplasma gondii* . Immunity 29: 306–317. 18691912 10.1016/j.immuni.2008.05.019 PMC2605393

[13] Robben PR, LaRegina M, Kuziel WA, Sibley LD (2005) Recruitment of Gr-1^+^ monocytes is essential for control of acute toxoplasmosis. J Exp Med 201: 1761–1769. 15928200 10.1084/jem.20050054 PMC2213275

[14] Zhao Y, Ferguson DJ, Wilson DC, Howard JC, Sibley LD, et al (2009) Virulent *Toxoplasma gondii* evade immunity-related GTPas-mediated parasite vacuole disruption within primed macrophages. J Immunol 182: 3775–3781. 19265156 10.4049/jimmunol.0804190 PMC2848815

[15] Wilson CB, Tsai V, Remington JS (1980) Failure to trigger the oxidative burst of normal macrophages. Journal of Experimental Medicine 151: 328–346. 7356726 10.1084/jem.151.2.328 PMC2185778

[16] Collazo CM, Yap GS, Sempowski GD, Lusby KC, Tessarollo L, et al (2001) Inactivation of LRG-47 and IRG-47 reveals a family of interferon gamma-inducible genes with essential, pathogen-specific roles in resistance to infection. J Exp Med 194: 181–188. 11457893 10.1084/jem.194.2.181 PMC2193451

[17] Taylor GA, Collazo CM, Yap GS, Nguyen K, Gregorio TA, et al (2000) Pathogen-specific loss of host resistance in mice lacking IFN-γ -inducible gene IGTP. Proc Nat Acad Sci 97: 751–755. 10639151 10.1073/pnas.97.2.751 PMC15402

[18] Ling YM, Shaw MH, Ayala C, Coppens I, Taylor GA, et al (2006) Vacuolar and plasma membrane stripping and autophagic elimination of *Toxoplasma gondii* in primed effector macrophages. J Exp Med 203: 2063–2071. 16940170 10.1084/jem.20061318 PMC2118399

[19] Martens S, Parvanova I, Zerrahn J, Griffiths G, Schell G, et al (2005) Disruption of *Toxoplasma gondii* parasitophorous vacuoles by the mouse p47-resistance GTPases. Plos Pathog 1: e24. 16304607 10.1371/journal.ppat.0010024 PMC1287907

[20] Bekpen C, Hunn JP, Rohde C, Parvanova I, Guethlein L, et al (2005) The interferon-inducible p47 (IRG) GTPases in vertebrates: loss of the cell autonomous resistance mechanism in the human lineage. Genome Biol 6: R92. 16277747 10.1186/gb-2005-6-11-r92 PMC1297648

[21] Shenoy AR, Kim BH, Choi HP, Matsuzawa T, Tiwari S, et al (2008) Emerging themes in IFN-gamma-induced macrophage immunity by the p47 and p65 GTPase families. Immunobiol 212: 771–784. 10.1016/j.imbio.2007.09.018 PMC2705969 18086378

[22] Taylor GA, Feng CG, Sher A (2007) Control of IFN-gamma-mediated host resistance to intracellular pathogens by immunity-related GTPases (p47 GTPases). Microb Infect 9: 1644–1651. 10.1016/j.micinf.2007.09.004 18023232

[23] Khaminets A, Hunn JP, Konen-Waisman S, Zhao YO, Preukschat D, et al (2010) Coordinated loading of IRG resistance GTPases on to the *Toxoplasma gondii* parasitophorous vacuole. Cell Microbiol 12: 939–961. 20109161 10.1111/j.1462-5822.2010.01443.x PMC2901525

[24] Fentress SJ, Behnke MS, Dunay IR, Moashayekhi M, Rommereim LM, et al (2010) Phosphorylation of immunity-related GTPases by a parasite secretory kinase promotes macrophage survival and virulence. Cell Host Microbe 16: 484–495. 10.1016/j.chom.2010.11.005 PMC3013631 21147463

[25] Steinfeldt T, Konen-Waisman S, Tong L, Pawlowski N, Lamkemeyer T, et al (2010) Phosphorylation of mouse immunity-related GTPase ( IRG) resistance proteins is an evasion strategy for virulent *Toxoplasma gondii* . PLoS Biol 8: e1000576. 21203588 10.1371/journal.pbio.1000576 PMC3006384

[26] Zhao Z, Fux B, Goodwin M, Dunay IR, Strong D, et al (2008) Autophagosome-independent essential function for the autophagy protein Atg5 in cellular immunity to intracellular pathogens. Cell Host Microbe 4: 458–469. 18996346 10.1016/j.chom.2008.10.003 PMC2682425

[27] Behnke MS, Khan A, Wootton JC, Dubey JP, Tang K, et al (2011) Virulence differences in *Toxoplasma* mediated by amplification of a family of polymorphic pseuodokinases. Proc Natl Acad Sci U S A 108: 9631–9636. 21586633 10.1073/pnas.1015338108 PMC3111276

[28] Reese ML, Zeiner GM, Saeij JP, Boothroyd JC, Boyle JP (2011) Polymorphic family of injected pseudokinases is paramount in *Toxoplasma* virulence. Proc Natl Acad Sci U S A 108: 9625–9630. 21436047 10.1073/pnas.1015980108 PMC3111280

[29] Reese ML, Boothroyd JC (2011) A conserved non-canonical motif in the pseudoactive site of the ROP5 pseudokinase domain mediates its effect on *Toxoplasma* virulence. J Biol Chem 286: 29366–29375. 21708941 10.1074/jbc.M111.253435 PMC3190742

[30] Boudeau J, Miranda-Saavedra D, Barton GJ, Alessi DR (2006) Emerging roles of pseudokinases. Trends Cell Biol 16: 443–452. 16879967 10.1016/j.tcb.2006.07.003

[31] Fox BA, Bzik DJ (2002) *De novo* pyrimidine biosynthesis is required for virulence of *Toxoplasma gondii* . Nature (London) 415: 926–929. 11859373 10.1038/415926a

[32] Yap GS, Sher A (1999) Effector cells of both nonhemopoietic and hemopoietic origin are required for interferon (IFN)-gamma- and tumor necrosis factor (TNF)-alpha- dependent host resistance to the intracellular pathogen, *Toxoplasma gondii* . J Exp Med 189: 1083–1091. 10190899 10.1084/jem.189.7.1083 PMC2192999

[33] Liesenfeld O, Parvanova I, Zerrahn J, Han SJ, Heinrich F, et al (2011) The IFN-gamma-inducible GTPase, Irga6, protects mice against Toxoplasma gondii but not against Plasmodium berghei and some other intracellular pathogens. PLoS One 6: e20568. 21698150 10.1371/journal.pone.0020568 PMC3117789

[34] Henry SC, Daniell XG, Burroughs AR, Indaram M, Howell DN, et al (2009) Balance of Irgm protein activities determines IFN-gamma-induced host defense. J Leukoc Biol 85: 877–885. 19176402 10.1189/jlb.1008599 PMC2669409

[35] Hunn JP, Koenen-Waisman S, Papic N, Schroeder N, Pawlowski N, et al (2008) Regulatory interactions between IRG resistance GTPases in the cellular response to *Toxoplasma gondii* . EMBO J 27: 2495–2509. 18772884 10.1038/emboj.2008.176 PMC2532785

[36] Feng CG, Weksberg DC, Taylor GA, Sher A, Goodell MA (2008) The p47 GTPase Lrg-47 (Irgm1) links host defense and hematopoietic stem cell proliferation. Cell Stem Cell 2: 83–89. 18371424 10.1016/j.stem.2007.10.007 PMC2278017

[37] Henry SC, Traver M, Daniell X, Indaram M, Oliver T, et al (2010) Regulation of macrophage motility by Irgm1. J Leukoc Biol 87: 333–343. 19920210 10.1189/jlb.0509299 PMC2812558

[38] Hanks SK, Hunter T (1995) Protein kinases 6. The eukaryotic protein kinase superfamily: kinase (catalytic) domain structure and classification. FASEB Journal 9: 576–596. 7768349

[39] Fleckenstein MC, Reese ML, Konen-Waisman S, Boothroyd JC, Howard JC, et al (2012) A *Toxoplasma gondii* pseudokinase inhibits host IRG resistance proteins. PLoS Biol 10: e1001358. 22802726 10.1371/journal.pbio.1001358 PMC3393671

[40] Fentress SJ, Sibley LD (2011) The secreted kinase ROP18 defends Toxoplasma's border. Bioessays 33: 693–700. 21773979 10.1002/bies.201100054 PMC3378050

[41] Melzer T, Duffy A, Weiss LM, Halonen SK (2008) The gamma interferon (IFN-gamma)-inducible GTP-binding protein IGTP is necessary for toxoplasma vacuolar disruption and induces parasite egression in IFN-gamma-stimulated astrocytes. Infect Immun 76: 4883–4894. 18765738 10.1128/IAI.01288-07 PMC2573374

[42] Yamamoto M, Ma JS, Mueller C, Kamiyama N, Saiga H, et al (2011) ATF6-beta is a host cellular target of the *Toxoplasma gondii* virulence factor ROP18. J Exp Med 208: 1533–1546. 21670204 10.1084/jem.20101660 PMC3135360

[43] Collazo CM, Yap GS, Hieny S, Caspar P, Feng CG, et al (2002) The function of gamma interferon-inducible GTP-binding protein IGTP in host resistance to *Toxoplasma gondii* is Stat1 dependent and requires expression in both hematopoietic and nonhematopoietic cellular compartments. Infect Immun 70: 6933–6939. 12438372 10.1128/IAI.70.12.6933-6939.2002 PMC132942

[44] Zeqiraj E, van Aalten DM (2010) Pseudokinases-remnants of evolution or key allosteric regulators? Curr Opin Struct Biol 20: 772–781. 21074407 10.1016/j.sbi.2010.10.001 PMC3014569

[45] Baas AF, Boudeau J, Sapkota GP, Smit L, Medema R, et al (2003) Activation of the tumour suppressor kinase LKB1 by the STE20-like pseudokinase STRAD. EMBO J 22: 3062–3072. 12805220 10.1093/emboj/cdg292 PMC162144

[46] Zeqiraj E, Filippi BM, Deak M, Alessi DR, van Aalten DM (2009) Structure of the LKB1-STRAD-MO25 complex reveals an allosteric mechanism of kinase activation. Science 326: 1707–1711. 19892943 10.1126/science.1178377 PMC3518268

[47] Zeqiraj E, Filippi BM, Goldie S, Navratilova I, Boudeau J, et al (2009) ATP and MO25alpha regulate the conformational state of the STRADalpha pseudokinase and activation of the LKB1 tumour suppressor. PLoS Biol 7: e1000126. 19513107 10.1371/journal.pbio.1000126 PMC2686265

[48] Niedelman W, Gold DA, Rosowski EE, Sprokholt JK, Lim D, et al (2012) The rhoptry proteins ROP18 and ROP5 Mediate *Toxoplasma gondii* evasion of the murine, but not the human, interferon-gamma response. PLoS Pathog 8: e1002784. 22761577 10.1371/journal.ppat.1002784 PMC3386190

[49] Puig O, Caspary F, Rigaut G, Rutz B, Bouveret E, et al (2001) The tandem affinity purification (TAP) method: a general procedure of protein complex purification. Methods 24: 218–229. 11403571 10.1006/meth.2001.1183

[50] Singh SB, Davis AS, Taylor GA, Deretic V (2006) Human IRGM induces autophagy to eliminate intracellular mycobacteria. Science 313: 1438–1441. 16888103 10.1126/science.1129577

[51] McCarroll SA, Huett A, Kuballa P, Chilewski SD, Landry A, et al (2008) Deletion polymorphism upstream of IRGM associated with altered IRGM expression and Crohn's disease. Nat Genet 40: 1107–1112. 19165925 10.1038/ng.215 PMC2731799

[52] Degrandi D, Konermann C, Beuter-Gunia C, Kresse A, Wurthner J, et al (2007) Extensive characterization of IFN-induced GTPases mGBP1 to mGBP10 involved in host defense. J Immunol 179: 7729–7740. 18025219 10.4049/jimmunol.179.11.7729

[53] Kim BH, Shenoy AR, Kumar P, Das R, Tiwari S, et al (2011) A family of IFN-gamma-inducible 65-kD GTPases protects against bacterial infection. Science 332: 717–721. 21551061 10.1126/science.1201711

[54] Virreira Winter S, Niedelman W, Jensen KD, Rosowski EE, Julien L, et al (2011) Determinants of GBP recruitment to *Toxoplasma gondii* vacuoles and the parasitic factors that control it. PLoS One 6: e24434. 21931713 10.1371/journal.pone.0024434 PMC3169597

[55] Yamamoto M, Okuyama M, Ma JS, Kimura T, Kamiyama N, et al (2012) A cluster of Interferon-gamma-inducible p65 GTPases plays a critical role in host defense against *Toxoplasma gondii* . Immunity 37: 302–313. 22795875 10.1016/j.immuni.2012.06.009

[56] Traver MK, Henry SC, Cantillana V, Oliver T, Hunn JP, et al (2011) Immunity-related GTPase M (IRGM) proteins influence the localization of guanylate-binding protein 2 (GBP2) by modulating macroautophagy. J Biol Chem 286: 30471–30480. 21757726 10.1074/jbc.M111.251967 PMC3162407

[57] Behnke M, Radke J, Smith AT, Sullivan WJ, White MW (2009) The transcription of bradyzoite genes in *Toxoplasma gondii* is controlled by autonomous promoter elements. Mol Microbiol 68: 1502–1518. 10.1111/j.1365-2958.2008.06249.x PMC2440561 18433450

[58] Pollock JD, Williams DA, Gifford MA, Li LL, Du X, et al (1995) Mouse model of X-linked chronic granulomatous disease, an inherited defect in phagocyte superoxide production. Nat Genet 9: 202–209. 7719350 10.1038/ng0295-202

[59] Mashayekhi M, Sandau MM, Dunay IR, Frickel EM, Khan A, et al (2011) CD8alpha(+) dendritic cells are the critical source of interleukin-12 that controls acute infection by *Toxoplasma gondii* tachyzoites. Immunity 35: 249–259. 21867928 10.1016/j.immuni.2011.08.008 PMC3171793

[60] Boyle JP, Saeij JP, Boothroyd JC (2007) *Toxoplasma gondii*: inconsistent dissemination patterns following oral infection in mice. Exp Parasitol 116: 302–305. 17335814 10.1016/j.exppara.2007.01.010

[61] Upadhya R, Kim K, Hogue-Angeletti R, Weiss LM (2011) Improved techniques for endogenous epitope tagging and gene deletion in *Toxoplasma gondii* . J Microbiol Methods 85: 103–113. 21352857 10.1016/j.mimet.2011.02.001 PMC3073369

[62] Charif H, Darcy F, Torpier G, Cesbron-Delauw MF, Capron A (1990) *Toxoplasma gondii:* characterization and localization of antigens secreted from tachyzoites. Exp Parasitol 71: 114–124. 2191870 10.1016/0014-4894(90)90014-4

[63] Dobrowolski JM, Niesman IR, Sibley LD (1997) Actin in the parasite *Toxoplasma gondii* is encoded by a single copy gene, *ACT1* and exists primarily in a globular form. Cell Motil Cytoskel 37: 253–262. 10.1002/(SICI)1097-0169(1997)37:3<253::AID-CM7>3.0.CO;2-7 9227855

[64] Dunn JD, Ravindran S, Kim SK, Boothroyd JC (2008) The T*oxoplasma gondii* dense granule protein GRA7 is phosphorylated upon invasion and forms an unexpected association with the rhoptry proteins ROP2 and ROP4. Infect Immun 76: 5853–5861. 18809661 10.1128/IAI.01667-07 PMC2583583

