# Supplementary figures and images for: The Polymorphic Pseudokinase ROP5 Controls Virulence in Toxoplasma gondii by Regulating the Active Kinase ROP18
Source: PLoS Pathog. 2012 Nov 8;8(11):e1002992. doi: 10.1371/journal.ppat.1002992 (PMC3493473; doi:10.1371/journal.ppat.1002992)

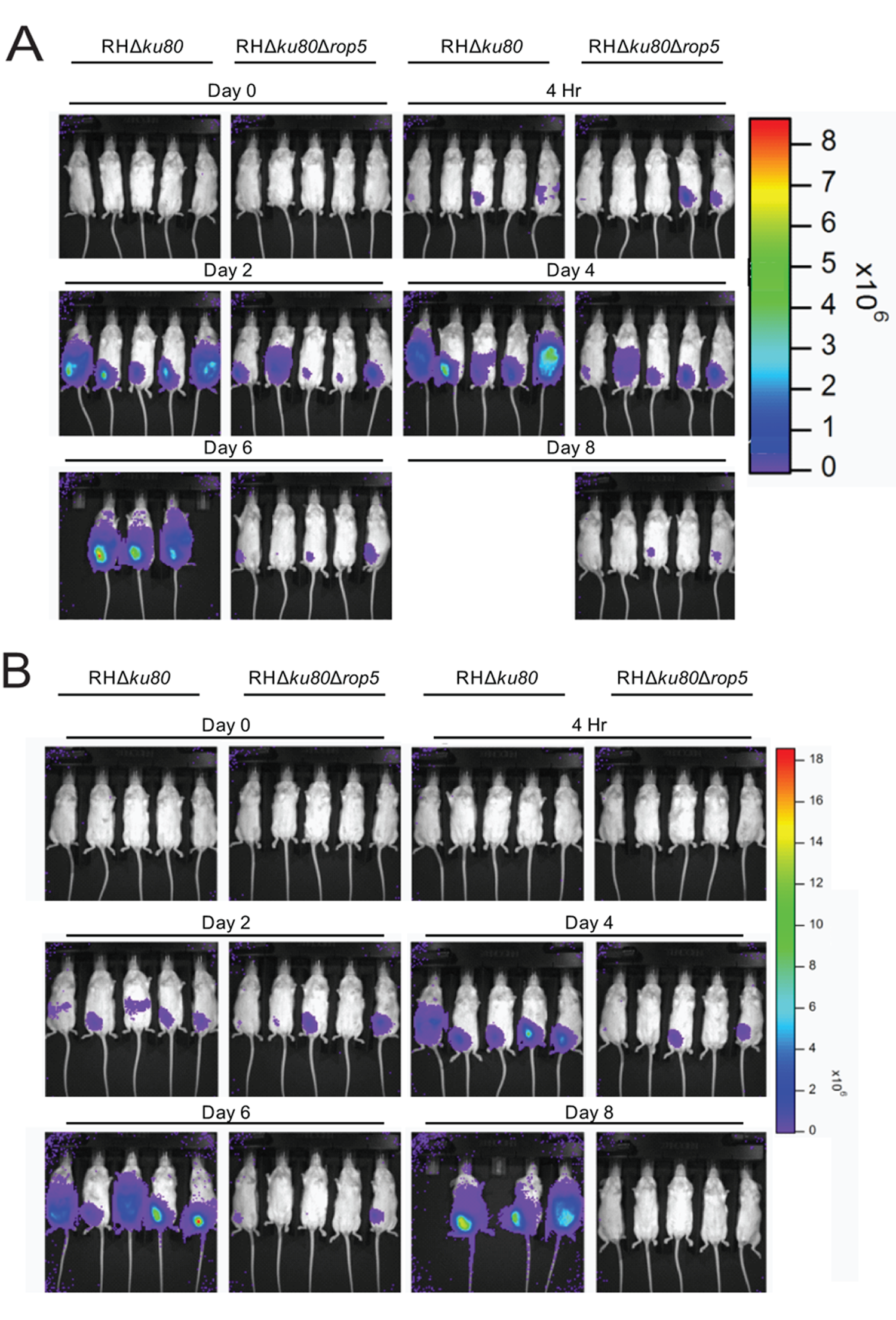

Supplement: Figure S1 — Luciferase imaging of infected mice. CD-1 mice infected with either 106 (A) or 103 (B) luciferase expressing wild type (RHΔku80) or ROP5 deficient (RHΔku80Δrop5) parasites, Day 0 through Day 8. Mice were injected i.p. with D-luciferin (Biosynth AG) at 150 mg/kg, anesthetized with 2% isoflurane for 5 min and imaged with a Xenogen IVIS 200 Imager and processed using Xenogen Living Image software (Caliper Life Sciences). Quantification of these images was used to graph data in Figure 1A. (TIF) [file ppat.1002992.s001.tif]

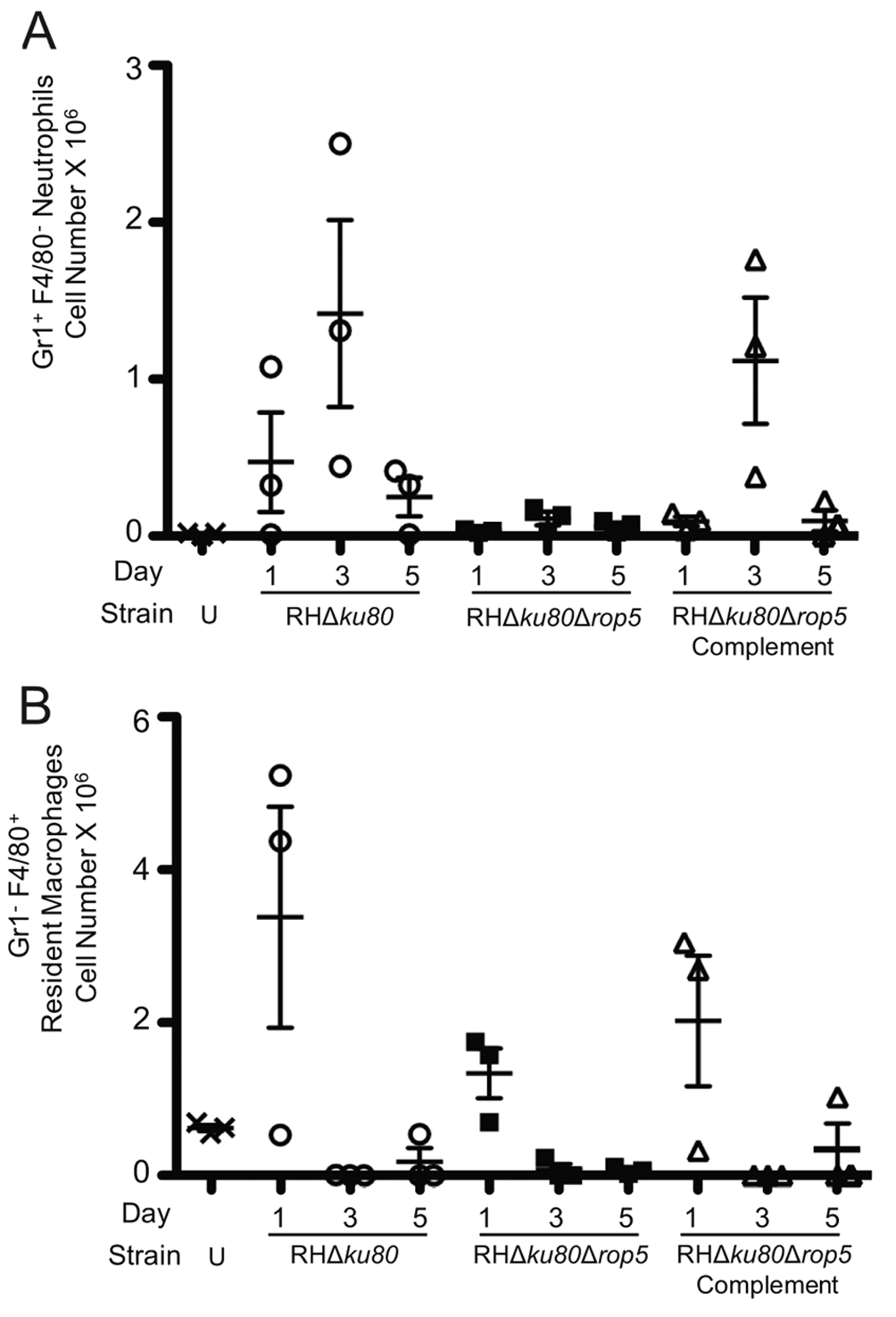

Supplement: Figure S2 — FACS analyses of cell populations in the peritoneum of challenged mice. (A) CD-1 mice were infected with 103 wild type (RHΔku80), ROP5 deficient (RHΔku80Δrop5), or ROP5 complemented (RHΔku80Δrop5Complement) parasites and the number of Gr1+ F4/80− cells (A, neutrophils) and Gr1− F4/80+ cells (B, resident macrophages) was determined by cell surface staining and FACS. Mean ± S.E.M., n = 3 animals per group. Representative experiment. Data in (A) and (B) were derived from the same cell populations as those in Figure 3A. (TIF) [file ppat.1002992.s002.tif]

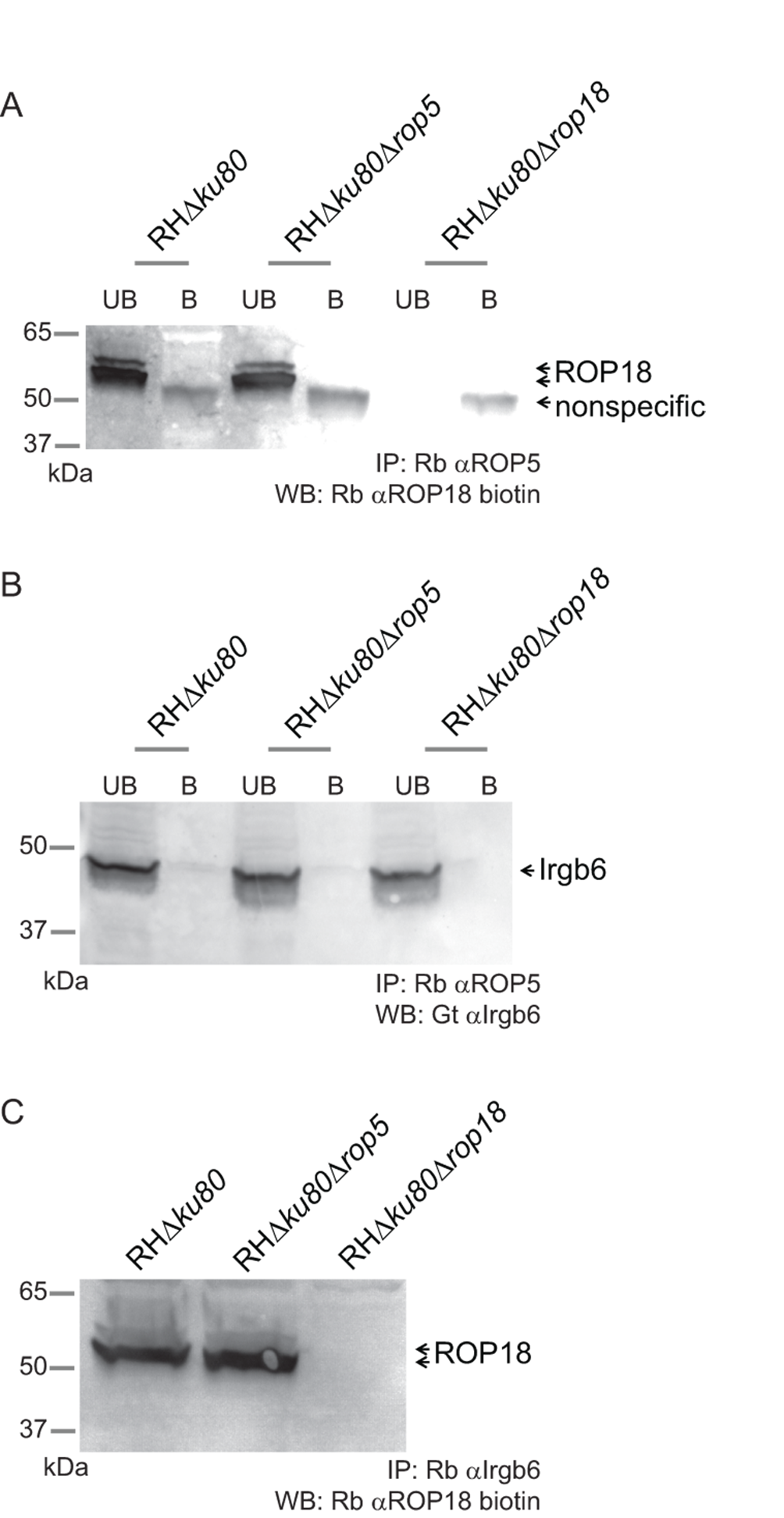

Supplement: Figure S3 — Irgb6-ROP18 protein interaction is present in the absence of ROP5. RAW cells were activated for 24 hr with 10 U/mL IFN-γ and 0.1 ng/mL LPS prior to infection for 30 min with indicated strains. (A) Immunoprecipitation of ROP5 from infected cell lysates with polyclonal rabbit anti-ROP5 (Rb αROP5). Unbound (UB) and bound (B) fractions were resolved by SDS-PAGE and blotted for ROP18 (Rb αROP18 biotin). ROP18 is denoted by the double arrows representing both full length and proteolytically processed protein. A third lower molecular weight nonspecific band present in all bound fractions likely represents heavy chain antibody. (B) Immunoprecipitation of ROP5 from infected cell lysates with polyclonal rabbit anti-ROP5 (Rb αROP5). Unbound (UB) and bound (B) fractions were resolved by SDS-PAGE and blotted with goat anti-Irgb6 (Gt αIrgb6, Santa Cruz A-20, denoted by arrow). (C) Immunoprecipitation of Irgb6 from infected cell lysates with polyclonal rabbit anti-Irgb6 (Rb αIrgb6). Bound (B) fractions were resolved by SDS-PAGE and blotted for ROP18 (Rb αROP18 biotin, denoted by double arrows). (TIF) [file ppat.1002992.s003.tif]

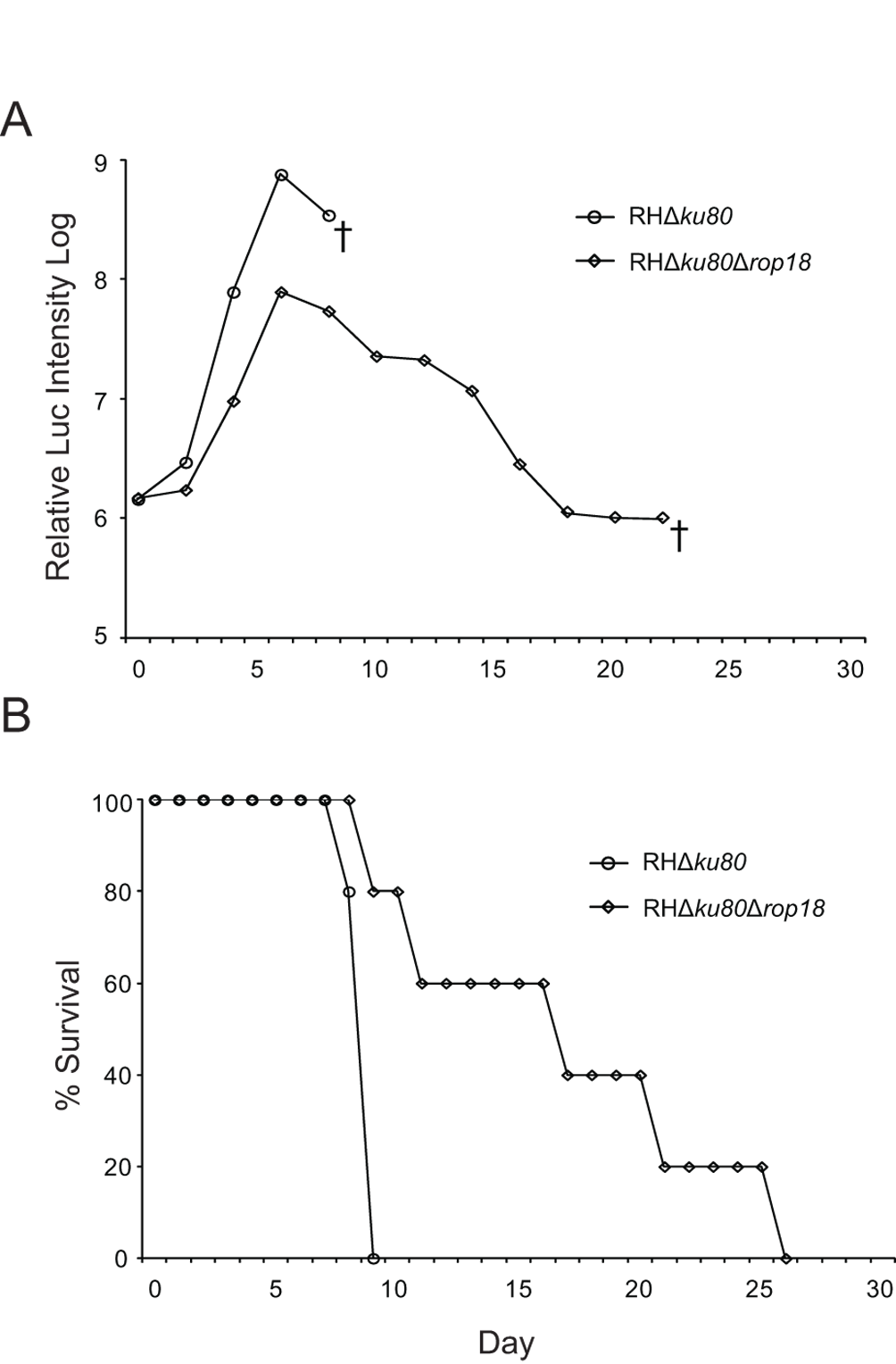

Supplement: Figure S4 — Luciferase imaging of RHΔ ku80 Δ rop18 infected CD-1 mice. CD-1 mice were i.p. injected with either 103 luciferase expressing wild type (RHΔku80) or ROP18 deficient (RHΔku80Δrop18) parasites and imaged on indicated days. † denotes one or more deaths. Mean values shown per group (n = 5). Representative experiment. (B) Survival curves for mice challenged in A. Representative experiment, n = 5 mice per group. Although luciferase signals returned to background levels in some mice before they expired, the presence of parasites in these mice could be detected with longer exposure, suggesting the mice eventually succumbed from low burden chronic infections. (TIF) [file ppat.1002992.s004.tif]

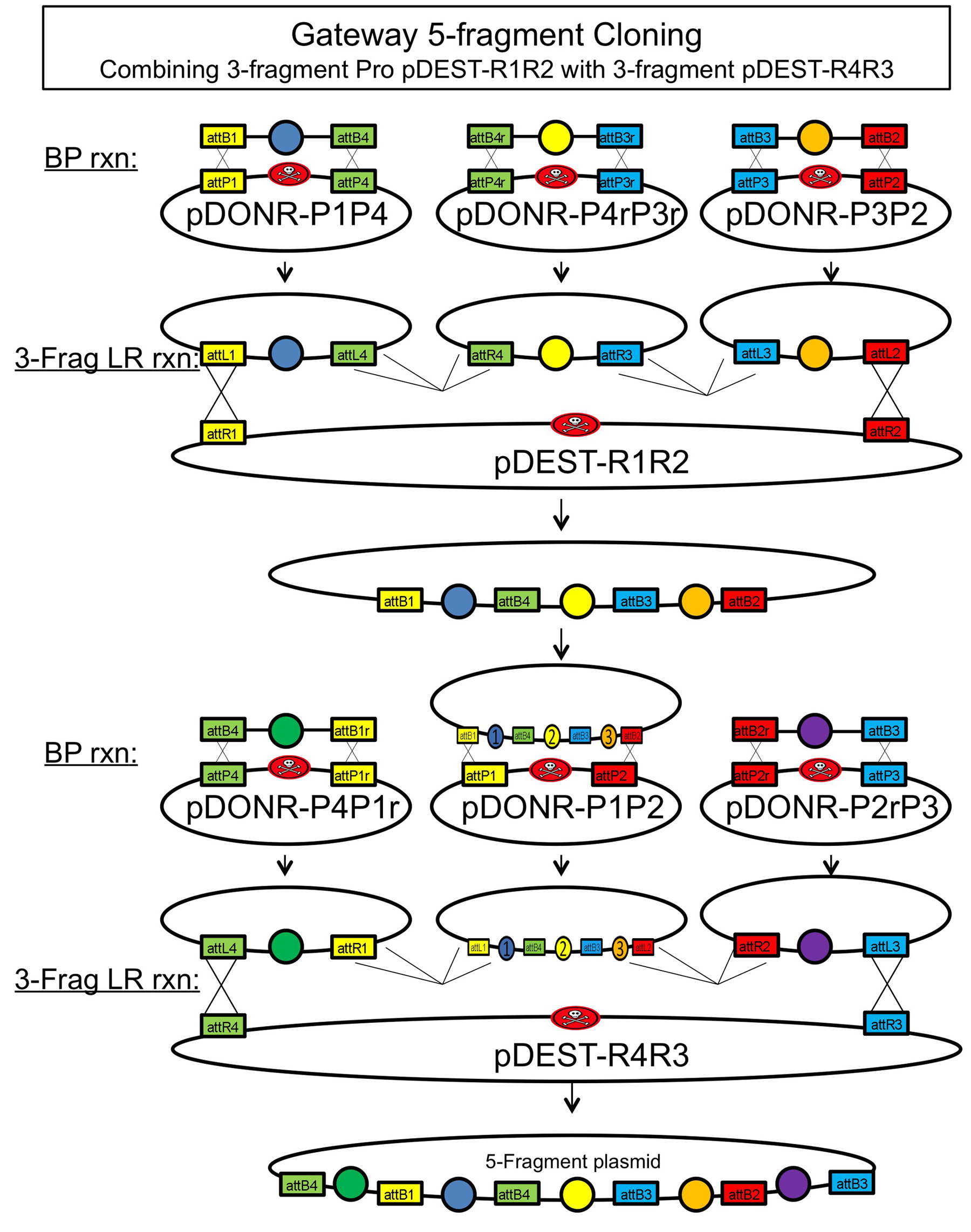

Supplement: Figure S5 — Gateway cloning construction of ROP18 complementation plasmid. The Gateway Cloning Technology (Invitrogen) is currently designed to allow for cloning of 1 to 4 separate fragments using site-specific recombinases (http://www.invitrogen.com/site/us/en/home/Products-and-Services/Applications/Cloning/Gateway-Cloning.html). We utilized currently available Gateway plasmids to construct a 5-fragment cloning strategy. By combining two 3-fragment MultiSite Gateway systems, pDEST-R1R2 and pDEST-R4-R3, we generated a 5-fragment plasmid that was used to complement ROP18. The middlemost cassette was first cloned into pDEST-R1R2 and used as the center fragment (pDONR-P1P2) in the second round of cloning into pDEST-R4R3. Although there are internal attB4 and attB3 sites in this center fragment, they do not hinder the ability to obtain a correct entry clone after the BP reaction with pDONR-P1P2 or an expression clone after the LR reaction with the 3-fragment pDEST-R4R3 system. To create the ROP18 complementation plasmid, we combined three fragments contained in separate plasmids: a genomic region 1,000 bp upstream of the IMC1 gene, assumed to contain its promoter, (i.e. TGME49_031630) (pDONR-P1P4); the ROP18 gene with a 3′ Ty tag (pDONR-P4rP3r); and the DHFR 3′ UTR (pDONR-P3P2) into the pDEST-R1R2 system. The IMC1 promoter and ROP18 gene were amplified from RH strain lysate using iProof High Fidelity DNA polymerase (Bio-Rad). The 3-fragment pDEST-R1R2 plasmid was used to clone the IMC1p-ROP18Ty-DHFR 3′ UTR fragment into pDONR-P1P2. This plasmid was then combined with the plasmids containing flanking regions of the UPRT gene used to construct pDestR4R3-UPRTKO-Clickluc (see Material and Methods) into the pDEST-R4R3 system to create the final 5-fragment plasmid referred to as pDEST-R4R3 (5-Frag)-UPRTKO-IMC1p-ROP18Ty-DHFR3′. Primers used for the various plasmids are found in Table S1. The fragment containing the UPRT 5′ KO-IMC1p-ROP18Ty-DHFR 3′ UTR- UPRT 3′ KO target sequence was PCR a [file ppat.1002992.s005.tif]

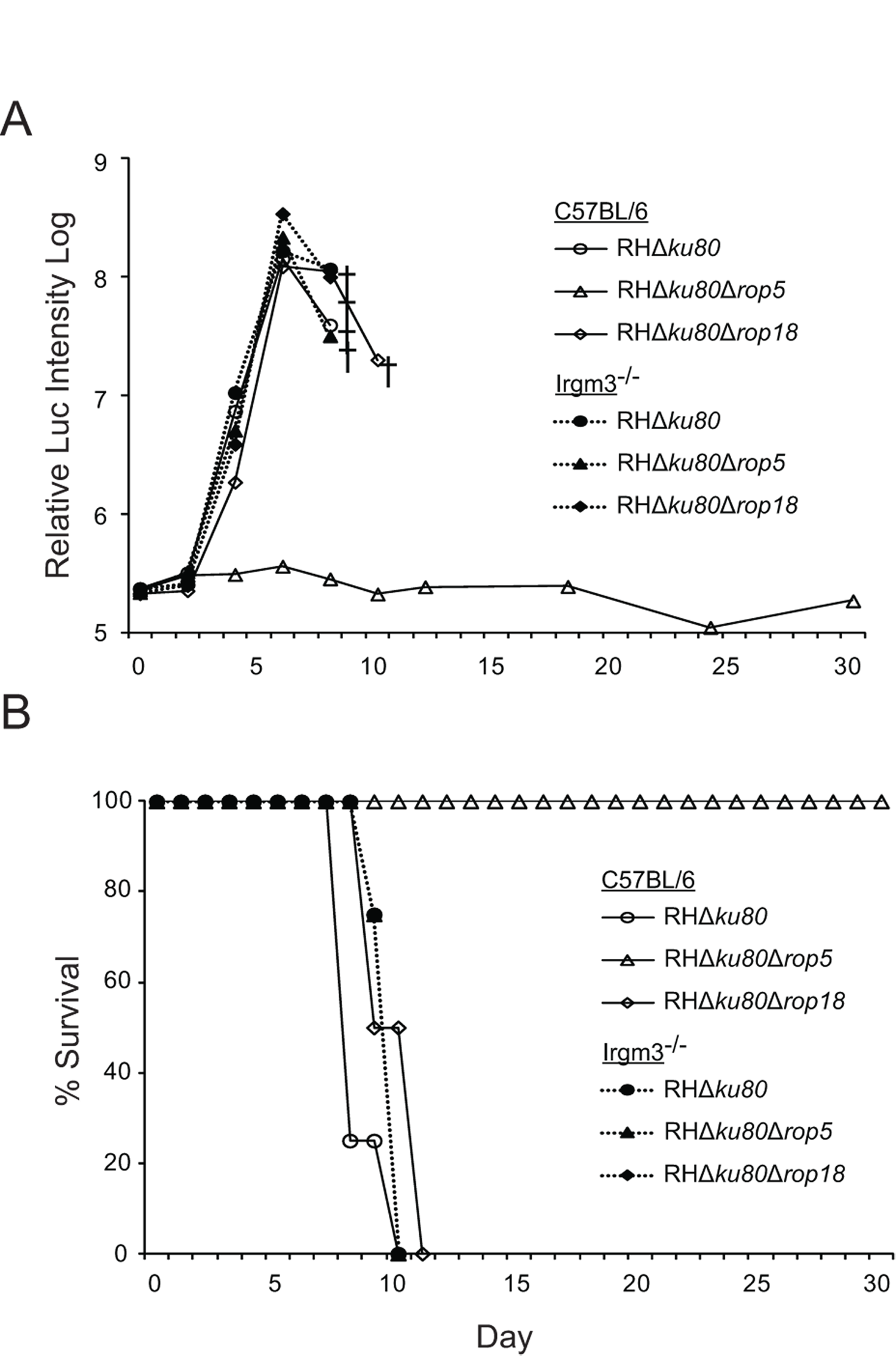

Supplement: Figure S6 — Luciferase imaging of i.p. injected Irgm3−/− mice. (A) Wild type (C57BL/6) (n = 4 per parasite strain) or Irgm3 deficient (Irgm3−/−) (n = 4 per parasite strain) mice were infected with 102 luciferase expressing wild type (RHΔku80), ROP5 deficient (RHΔku80Δrop5), or ROP18 deficient (RHΔku80Δrop18) parasites by i.p. injection and imaged on indicated days. † denotes one or more death per group. Representative experiment. (B) Survival curves for mice challenged with T. gondii strains as shown. Combination of two experiments (n = 8 animals per group). (TIF) [file ppat.1002992.s006.tif]
