# Supplementary material for: The Polymorphic Pseudokinase ROP5 Controls Virulence in Toxoplasma gondii by Regulating the Active Kinase ROP18
Source: PLoS Pathog. 2012 Nov 8;8(11):e1002992. doi: 10.1371/journal.ppat.1002992 (PMC3493473; doi:10.1371/journal.ppat.1002992)
Supplement: Table S1 — Primers used in this study. (PDF) [file ppat.1002992.s007.pdf]

Table S 1 Primers used in this study

Primer Name	Primer	Use
attB1 DHFR promoter - forward (amplified DHFR 5'-Clickluc-DHFR 3')	5' -GGGGACA AGTTTGTACAAAAAAGCAGGCTTCCAGCACGAAACCTTGCATTCA- 3'	pDestR4R3-UPRTKO-Clickluc
attB2 DHFR 3' UTR - reverse (amplified DHFR 5'-Clickluc-DHFR 3')	5' -GGGGACCACTTTGTACAAGAAAGCTGGGTCTTCAATGATCCCCCTCCACC- 3'	pDestR4R3-UPRTKO-Clickluc
attB1 IMC1 promoter - forward	5' -GGGGACAAGTTTGTACAA AAAGCAGGCTGTGCCAGCGATGAACAGCCA- 3'	pDEST-R4R4(5-Frag)-UPRTKO-IMC1p-ROP18Ty-DHFR3'
attB4 IMC1 promoter - reverse	5' -GGGGACAACTTTGTATAGAAAAGTTGGGTGGGTGAGAAAGGAGGGAACAACT- 3'	pDEST-R4R4(5-Frag)-UPRTKO-IMC1p-ROP18Ty-DHFR3'
attB4r ROP18 cds - forward	5' -GGGGACAACTTTTCTATACAAAGTTGTCATGTTTTCGGTACAGCGGCC- 3'	pDEST-R4R3(5-Frag)-UPRTKO-IMC1p-ROP18Ty-DHFR3'
attB3r ROP18 cds with Ty tag - reverse	5'GGGGACAACTTTATTATACAAAGTTGTTTAATCGAGCGGGTCCTGGTTCGTGTGGA CCTCTTCTGTGTGGAGATGTTC- 3'	pDEST-R4R4(5-Frag)-UPRTKO-IMC1p-ROP18Ty-DHFR3'
attB3 DHFR 3' UTR - forward	5' -GGGGACA ACTTTGTATAATAAAGTTGTCGGAGAGAGCTGACGAGGGGGT- 3'	pDEST-R4R4(5-Frag)-UPRTKO-IMC1p-ROP18Ty-DHFR3'
attB2 DHFR 3' UTR - reverse	5' -GGGGACCACTTTGTACAAGAA AGCTGGGTACCGCGGTGTCACTGTAGCC- 3'	pDEST-R4R4(5-Frag)-UPRTKO-IMC1p-ROP18Ty-DHFR3'
attB4 UPRT 5' KO - forward	5' -GGGGACAACTTTGTATAGAAAAGTTGTCCCGCTGTGCCTAGTATCG- 3'	pDEST-R4R3-UPRTKO-Clickluc & pDEST-R4R3(5-Frag)-UPRTKO-IMC1p-ROP18Ty-DHFR3'
attB1r UPRT 5' KO - reverse	5' -GGGGACTGCTTTTTTGTACAAACTTGTTTTAGAAGCCCTGTGGA- 3'	pDEST-R4R3-UPRTKO-Clickluc & pDEST-R4R3(5-Frag)-UPRTKO-IMC1p-ROP18Ty-DHFR3'
attB2r UPRT 3' KO - forward	5' -GGGGACAGCTTTCTTGTACAAAGTGGTCGTCTCTAGTTTTTTTGACAGAC- 3'	pDEST-R4R3-UPRTKO-Clickluc & pDEST-R4R3(5-Frag)-UPRTKO-IMC1p-ROP18Ty-DHFR3'
attB3 UPRT 3' KO - reverse	5' -GGGGACAACTTTGTATAATAAAGTTGTCGACGTCAACTGTACGAC - 3'	pDEST-R4R3-UPRTKO-Clickluc & pDEST-R4R3(5-Frag)-UPRTKO-IMC1p-ROP18Ty-DHFR3'
PCR amplification of target construct - UPRT 5' KO region - forward	5' -GCTGTAGAGTTGCCACCC- 3'	to create PCR product for use in parasite transfection
PCR amplification of target construct - UPRT 3' KO region - reverse	5' -GTACGACATCCGAGTGAA- 3'	to create PCR product for use in parasite transfection
